# Vultures and Livestock: The Where, When, and Why of Visits to Farms

**DOI:** 10.3390/ani10112127

**Published:** 2020-11-16

**Authors:** Marina García-Alfonso, Thijs van Overveld, Laura Gangoso, David Serrano, José A. Donázar

**Affiliations:** 1 Department of Conservation Biology, Estación Biológica de Doñana (CSIC), C/Américo Vespucio 26, 41092 Sevilla, Spain; mathijs.van.overveld@gmail.com (T.v.O.); serrano@ebd.csic.es (D.S.); donazar@ebd.csic.es (J.A.D.); 2 Institute for Biodiversity and Ecosystem Dynamics, University of Amsterdam, Science Park 904, 1098 XH Amsterdam, The Netherlands; laurag@ebd.csic.es

**Keywords:** biodiversity conservation, Egyptian Vultures, foraging behavior, GPS tracking, livestock farms, supplementary feeding stations, Canary Islands

## Abstract

**Simple Summary:**

The abandonment of carcasses around livestock farms has been recently legalized in Europe. Since little is known about how vultures use this kind of resource, we aimed to determine the main drivers of vultures’ visits to farms. We evaluated the effects of characteristics of both birds and farms regarding the way that vultures visit farms thanks to data collected from 45 GPS-tagged Egyptian Vultures and most farms on Fuerteventura Island, Spain (318 farms with >94% of insular livestock). We found that farms were more visited when they were located close to highly predictable feeding places, when they had more available food, and during the vulture breeding season, whereas farms located close to roads and vultures’ breeding territories received fewer visits. Younger territorial birds visited a farm more frequently than older territorial ones, whereas older non-territorial individuals concentrated those visits on farms closer to their main centers of activity compared with younger ones. Our findings indicate that visits to farms were determined by their spatial distribution regarding bird activity centers, availability of carcasses, seasonality, and vulture characteristics. Hence, these factors should be considered in vulture conservation, avoiding very general solutions that ignore population structure and that could be not enough to protect the biodiversity.

**Abstract:**

Recent changes in European legislation have legalized the abandonment of carcasses around livestock farms, but our understanding of how vultures exploit these semi-predictable food sources is still very limited. For filling this gap, we determine the individual and ecological drivers influencing vulture visits to farms. We assessed the effects of individual characteristics of both birds and farms on the frequency of vultures’ visits to livestock facilities using data collected from 45 GPS-tagged Egyptian Vultures (*Neophron percnopterus*) and 318 farms (>94% of livestock) on Fuerteventura Island, Spain. Farms were more visited during the vultures’ breeding season. Farms located closer to highly predictable feeding places (i.e., vulture restaurants and garbage dumps) or with more available feeding resources were visited by more vultures, whereas those located close to roads and vultures’ breeding territories received fewer visits. Younger territorial birds visited a farm more frequently than older territorial ones, whereas older non-territorial individuals concentrated those visits on farms closer to their activity core areas compared with younger ones. Our findings indicate that visits to farms were determined by their spatial distribution in relation to the age-specific birds’ activity centers, the availability of carcasses, seasonality, and individual characteristics of vultures. These interacting factors should be considered in vulture conservation, avoiding very general solutions that ignore population structure.

## 1. Introduction

The abandonment of livestock carcasses near livestock facilities, as opposed to the use of feeding stations, is generally considered the more suitable strategy to preserve populations of endangered scavengers in traditional agro-grazing systems [1]. Farms are more numerous and widely distributed than would be feasible for any network of feeding stations. In addition, livestock deaths and therefore food provision occurs sporadically, in contrast to the regular disposal of slaughterhouse remains. This pattern may reduce the spatiotemporal predictability of food sources (i.e., creating a semi-predictable resource), thus favoring less competitive (small-sized) and often more endangered species and populations of avian scavengers [2,3,4]. Carcass abandonment at farms has additional economic and environmental advantages, mainly through the ecosystem service provided by scavengers that may largely reduce costs for carcass removal while also reducing associated pollution [5,6]. Scientists and conservation managers raised serious concerns about the potentially devastating effects of European sanitary regulations (i.e., mandatory destruction of livestock carcasses, EC1774/2002) on Old World vulture populations [7]. Consequently, EU legislation has shifted to allow the abandonment of carcasses outside feeding stations (CE 322/2003, CE 830/2005 CE 142/2011) [8], including in the vicinity of farms in some cases [9]. However, since no mandatory guidelines exist, each state member can develop its own regulations [9]. Although this initiative has been received with optimism and some general guidelines have been drafted [10,11,12], there is a general lack of scientific knowledge to deal with this emerging scenario.

Evidence suggests that individual bird characteristics such as age, sex, breeding stage, and social rank may explain asymmetries between conspecifics in the exploitation of predictable food resources [13]. This indicates that patterns of individual resource use within vulture populations can be more complex than generally acknowledged. Despite the belief that semi-predictable feeding conditions (e.g., those found at farms) may be more beneficial than highly predictable ones (e.g., feeding stations) [2,3,4,10,11,12], precise knowledge on farm use by vultures is still lacking.

Here, we aim at filling this important gap by focusing on the identification of ecological or individual-level drivers that influence visits to livestock farms by Egyptian Vultures (*Neophron percnopterus*) on Fuerteventura, Canary Islands. Our study system is particularly suitable to address this issue because the Canarian population of Egyptian Vulture, entirely comprised by the endemic subspecies *N. p. majorensis*, is threatened and subject to management actions, such as the creation of supplementary feeding stations. This subspecies subsists fundamentally on the island of Fuerteventura, which still holds a high number of goat farms under extensive regime [14,15]. Currently, livestock farming is shifting to semi-intensive and intensive practices, and the number of small traditional farms is declining. Intensive practices involve high concentrations of confined livestock and a more intensive use of antimicrobials [16]. The new European sanitary regulations have not yet been applied on the Canary Islands such that the abandonment of livestock remains near farms is still illegal. However, carcass abandonment frequently occurs, especially at farms located in remote areas [17].

The Canarian population of Egyptian Vultures has been intensively monitored over two decades. We used GPS data from 45 Egyptian Vultures tagged between 2013 and 2016 to examine patterns of visits to farms. We investigated factors related not only to farm attributes (e.g., size and location), but also far less explored variables related to individual characteristics, such as sex, age, breeding status, and activity areas. For this purpose, we followed a dual approach from the point of view of (i) farm characteristics and (ii) individual asymmetries. Understanding how the characteristics of both livestock exploitations and individual birds shape patterns of resource use by vultures is essential to address the challenge of their long-term conservation in a rapidly changing rural landscape. 

Within this context, we formulated a number of specific predictions that are detailed in Table 1. In general, we expect that vultures will be attracted to farms with higher quantity and predictability of food resources, and less human disturbance. We also expect to find differences in the use of farms by vultures related to sex- and age-dependent spatial and temporal constraints (see Table 2 for the description of the variables considered). 

## 2. Materials and Methods

### 2.1. Field Procedures

Fieldwork was conducted on Fuerteventura, the easternmost island of the Canary archipelago. The Canary Islands are situated in the north-east Atlantic Ocean, between 27°37′ and 29°25′ N, and 13°20′ and 18°10′ W, being Fuerteventura (1660 km^2^) the south-easternmost island. The landscape is semi-arid, dominated by grass and scrublands with an almost total absence of woodland [24]. Farming is based on livestock (goats and, to a lesser extent, sheep [15]). Goats were introduced with human colonization of the island around 3500 BP, remaining through the centuries as the main economic activity of the island [15]. Traditionally, the exploitations of goats on a small scale and in a semi-extensive regime have been the most typical practice in Fuerteventura, but in recent decades, the intensification of the exploitations has gained ground, as observed elsewhere [25]. The number of heads increased from 20,000 in 1970 to 155,000 in 2006, but it decreased from 2013 onwards [26].

**Figure 1:**
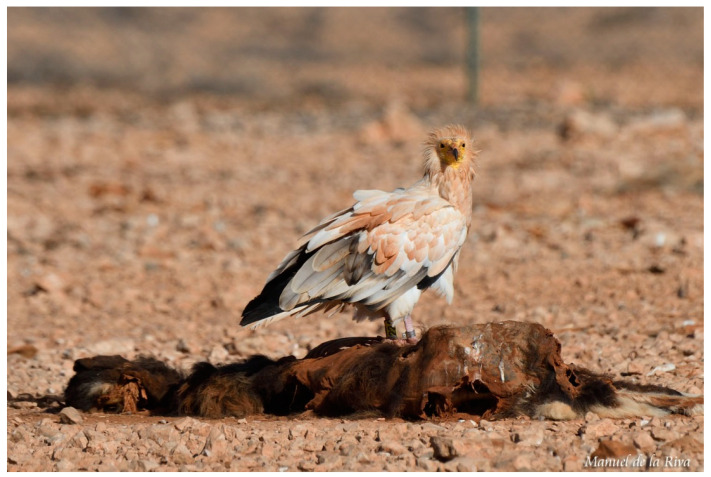
Canarian Egyptian Vulture feeding on a goat carcass in the supplementary feeding station located in the center of Fuerteventura Island. Photography© Manuel de la Riva.

The Canarian Egyptian Vulture (Figure 1) is a medium-sized (2–3 kg) and long-lived territorial scavenger whose population strongly declined during the 20th century owing to the incidence of non-natural mortality, mainly accidents with power lines and indirect poisoning [27]. Currently, it survives only in the eastern islands with the bulk of the population concentrated in Fuerteventura. The species has deferred sexual maturity with an age of recruitment between 4 and 10 years in the Canarian population [28]. The breeding season lasts from early February to late June. Throughout the year, but especially during the non-breeding season, Egyptian Vultures congregate in large numbers at communal roosts located on power lines as well as at predictable feeding places such as the garbage dump, feeding stations, and large livestock farms (see [13,27] for details).

**Figure 2:**
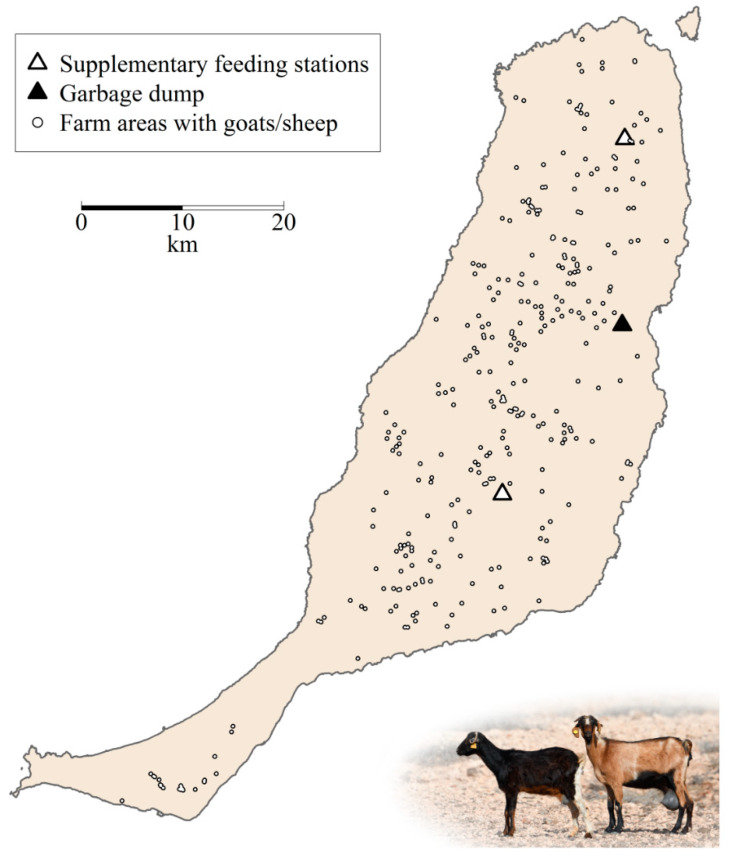
Farm locations and areas resulting after using a buffer of 180 meters around the location of each goat or sheep farm present on Fuerteventura (Canary Islands) from 2013 to 2016. Triangles show the location of highly predictable feeding places (HPFP). Photography© Manuel de la Riva.

This vulture population is sedentary and endemic to the archipelago [29] where it strongly depends on goat carcasses, which account for up to 79% of its diet [14,15]. Apart from food obtained at farms, vultures exploit resources (goat carcasses and slaughterhouse remains, mainly pig heads and viscera) provided at two supplementary feeding stations and other leftovers at a large garbage dump (Figure 2, see also [13]). Natural available food resources (e.g., small/medium-sized carrion such as rabbits, rodents, and pigeons, but also feral goats) also constitute an important component of their diet [14,30].

This subspecies has been intensively monitored since 1998. Intensive marking schemes (metallic and plastic rings) have determined that about 90% of the population was individually identifiable in 2018 [31]. Territories were regularly visited to identify breeding birds and to record breeding parameters.

From June 2013 to September 2016, we trapped 45 Canarian Egyptian Vultures (22 males and 23 females, aged from 0 (recently fledged young) to 14 years old, Table S1) with cannon-nets to equip them with solar-powered GPS transmitters. This accounted for 16% of the total population size estimated in 2016 [13]. Two types of GPS devices were used: 27 individuals were equipped with UvA-BiTS [32], 17 were equipped with E-obs devices (GmbH, Munich, Germany), and one individual successively carried a device of each type. Both kinds of devices have multiple onboard sensors providing the geographical coordinates, altitude, and speed of each individual according to a defined time interval (see below). Devices were attached as backpacks using 0.84 and 1.12 cm wide Teflon harnesses. The total system weight was between 31 g (UvABiTS) and 54 g (E-obs), respectively about 1.4–2.4% of the mean body mass, which is below the limit recommended by previous studies to avoid negative effects (3%, see [33]).

We used GPS data from 1 July 2013 to 1 January 2017. For vultures tagged before 2015 (*n* = 21, Table S1), the time interval between locations varied from 3 s to 20 min due to initial tests of the functioning of the devices. From 2015 onwards, all devices were programmed with time intervals between 1 and 5 min, with 97.5% location fixes recorded at time intervals lower than 350 s (Figure S1).

The ethical approval for this study was provided by “Comisión de Ética en la Investigación Experimental de la Universidad Miguel Hernández” with the code DBA-JSZ-001-12. Field procedures were carried out under the Project License approved by The Biodiversity Directorate of the Government of the Canary Islands (permit numbers: 2014/256, 2015/1652, and 2016/1707).

### 2.2. Characteristics of Farms

We gathered information on the geographical location and number of livestock (goats and sheep) from 318 farms on Fuerteventura between 2013 and 2016 (Figure 2). Data were obtained from both the Spanish Government [34] and from unpublished information from regional and local Governments (Dirección General de Protección de la Naturaleza of the Canarian Government and Cabildo Insular de Fuerteventura). We completed this information with 122 personal phone calls to local farmers (each owning >100 goats or sheep) to obtain information (or get confirmation) about their locations and number of livestock. As a result, between 2013 and 2016, we knew the location and herd size of 280–306 farms holding 94–98% of the total livestock on the island. This figure varied between 85,000 and 102,000 heads, around 85% of them being goats and the remaining sheep.

Although animal remains are regularly collected by a carcass-collecting service and buried in a garbage dump, some carcasses are still abandoned in the field and thus made available to vultures. Through the phone calls, we also gathered information on carcass abandonment by the farmers. From a total of 94 farmers, 57 indicated they usually abandoned carcasses around their farms (despite this being illegal, Regulation CE 1774/2002). Of the 37 who did not, 8 carried carcasses to the garbage dump, 3 carried carcasses to supplementary feeding stations, 21 used the carcass-collecting service, 2 buried them, 1 carried them to a nearby mortuary, and 2 stated that they never had carcasses. This information was used to predict values for those farms with unavailable information (see Section 2.4 below).

### 2.3. Use of Farms by Vultures

A first step was to characterize the distribution of dumping sites used by farmers in relation to the distance to the main building of their farming operations. Based on data from 10 interviews, i.e., all farmers who revealed the exact location of their illegal dumping sites, this gave a mean distance of 286 m (95% CI: 180–393 m). We used the lower 95% CI limit (180 m) as a buffer area in which the presence of vultures would be likely associated with feeding activities within the farm. If buffers of neighboring farms overlapped, we considered them as a unique farm area. Following this criterion, we grouped 318 farms into 260 farm areas, which are considered as “farms” hereafter. Within the above-mentioned CI limits for determining buffer areas, we decided to use the minimum value to minimize the combination of farms in the same “farm area” because they could have a different number of livestock heads as well as different “farmer behavior” in relation to carcass abandonment. Nevertheless, the analytical approach using the upper CI limit led to qualitatively similar patterns (results not shown).

Subsequently, we determined vultures’ visits to farms by using GPS data. We considered as “visits” those GPS fixes recorded inside each farm (i.e., farm area) which indicated that the bird would have landed: flight altitude lower than 25 m (Digital Elevation Model provided by [35]) and an instantaneous speed lower than 2 m/s (speed selected as on [17,36]). Field observations from the intensive monitoring carried out with this vulture population indicate that Egyptian Vultures search for food and forage at farms. Consequently, we considered that visits are reliable indicators of the use of these exploitations for feeding purposes, regardless of whether or not vultures obtained food in each particular visit.

### 2.4. Statistical Analyses

Since vulture behavior depends on breeding status and season [13], data were analyzed at a six-month interval (*Semester ID*), so that the first semester of each year was considered as the breeding season (*breeding* = 1) and the second was considered as the non-breeding season (*breeding* = 0). We used these seasons instead of shorter periods because the phenology of the target population is highly variable, with laying dates extending over several months (January to April). Consequently, precisely determining the periods corresponding to the different breeding tasks (e.g., incubation, chick rearing) would make the analyses unnecessarily complex, especially because central-place foraging constraints operate within both reproductive stages. We only included those vultures–semesters with available GPS information for at least 15 days per month (Table S1) and only quantified visits to active farms (some farms closed during the study period).

We established a double analytical approach to assess the factors determining variation in (i) the number of vultures visiting each farm, based on the number of tracked vultures, and (ii) the individual use of each farm, based on the number of visits per individual. Consequently, we used the following response variables:

(1) *FARM*: number of different individual vultures present at each farm during each semester, controlling for the total number of individuals with available information for that period.
(2) *VULTURE*: number of days each vulture visited each farm per semester, controlling for the total number of days with available information for that vulture during that semester. Since breeding activities may affect the use of particular feeding sources, we performed separate analyses on *territorial* (i.e., individuals showing territorial behaviour) and *non-territorial* vultures. For each individual, we quantified home ranges by using 95% kernel density estimates (KernelUD 95%, smoothing factor = 750, see [13] for details) and considered all farms located inside its home range as potential food sources.

Explanatory variables are described in Table 2. The variable *Carcass* was calculated using the information about the abandonment of carcasses obtained by phone calls and the predicted values of a Generalized Linear Model (GLM) fitted with this information and applied to farms for which we lacked information (*n* = 183) (see Section 2.4.1 below for a detailed description of the procedure). Response variables were modeled as proportion data with a binomial denominator by means of Generalized Linear Mixed Models (GLMMs) with binomial error distribution and logit link function. *Farm ID* and *Semester ID* were included as random factors in *FARM* models, while *Farm ID*, *Semester ID,* and *Bird ID* were included as random factors in *VULTURE* models (Table 2), according to improvements in Akaike’s Information Criterion corrected for small sample sizes (AICc) values ([37,38], see Tables S2–S4 for details). We standardized all continuous explanatory variables to a mean of 0 with a variance of 1 by subtracting the means and dividing by the standard deviations [39,40,41]. Models were fitted with all possible combinations of explanatory variables (from only the intercept to all considered fixed effects), except for pairs of variables with a Spearman’s correlation coefficient higher than |0.5| that were never included in the same model to avoid collinearity problems [42]. Collinearity was additionally checked using the variance inflation factor (VIF), which showed VIF values close to 1 for all predictors included in models (Table S5). Additionally, we fitted models including only one interaction at each time, considering all two-way interactions of biological interest. Model selection was done on the basis of the AICc. We discarded models including uninformative parameters, i.e., additional variables in top-ranked models not explaining sufficient deviance to provide a net reduction in AICc [43,44]. We tested for overdispersion [45] and determined the pseudo-R squared [46] in the selected models as a goodness of fit. To deal with model selection uncertainty, when there was no clearly supported model (Akaike weight ≥ 0.9), we performed model averaging of those top-ranked models with Akaike weights >0.001 [44].

Spatial autocorrelation in model residuals was checked using spline correlograms and Moran’s Index (Moran’s I) with nearest neighbors [47,48]. We used R statistical software version 3.4.0 [49] with the stats package for confidence intervals, lme4 [50] for the GLMM analysis, glmmTMB [51] for additional GLMM analysis, AICcmodavg [52] for model ranking, MuMIn [53] for calculating pseudo-R squared, usdm [54] for calculating VIF, modEvA for calculating adjusted explained deviance [55], and RVAideMemoire [56] for calculating overdispersion in GLMMs.

#### 2.4.1. Carcass Models

Since we had very limited information on carcass abandonment by farmers (see above), we estimated its probability of occurrence in each farm (variable *Carcass* in Table 2) by applying the results of a GLM fitted for the farms in which this variable was known (*n* = 77 farms). In this GLM (binomial error, logit link function), the response variable was whether carcasses were abandoned or not, and the explanatory variables were as follows: (a) *Dist Dump,* calculated as the distance from each farm to the insular garbage dump, and included because some farmers carried carcasses to the dump; (b) *Dist AFS,* calculated as the distance from each farm to the nearest artificial feeding station, and included because some farmers carried carcasses there; (c) *Dist Road*, calculated as the distance from the centroid of each farm to the nearest road, because difficulties in accessing farms influence the availability of the carcass-collecting service; (d) *Goat Sheep*, the number of goats and sheep on the basis of livestock censuses for each farm and semester, because large farms produce more remains, thus making abandonment more problematic; (e) *Dist Urb*, calculated as the distance from the centroid of each farm to the nearest urban area, because farms located inside or very close to urban areas can rarely abandon carcasses without detection. All distances were measured in meters.

Models were fitted with all possible combinations of explanatory variables, except for pairs of variables with a Spearman’s correlation coefficient higher than |0.5|, which were never included in the same model to avoid collinearity problems [41]. Model selection was carried out on the basis of the AICc [42]. We discarded models including uninformative parameters [43,44] and determined the adjusted explained deviance [57]. The top-ranked model selected to calculate the variable *Carcasses* (Table S6) indicated that the probability of carcass abandonment increased with the distance to both urban areas and the garbage dump on the island (Table S7).

## 3. Results

### 3.1. General Patterns

Overall, males visited more different farms than females, although differences also depended on the individual breeding status and seasonality. Territorial birds of both sexes included a similar number of farms within their home ranges, while in non-territorial individuals, males included more farms than females. Non-territorial birds visited more different farms during the breeding season, whereas territorial birds visited a similar number in both seasons (see details of statistical test on Table 3 and values of the variables on Figure S2). In fact, within non-territorial individuals, there was a strong correlation between the number of farms visited and the individual home range size (Spearman’s correlation coefficient = 0.82, *n* = 108, *p* < 0.001).

**Figure 3:**
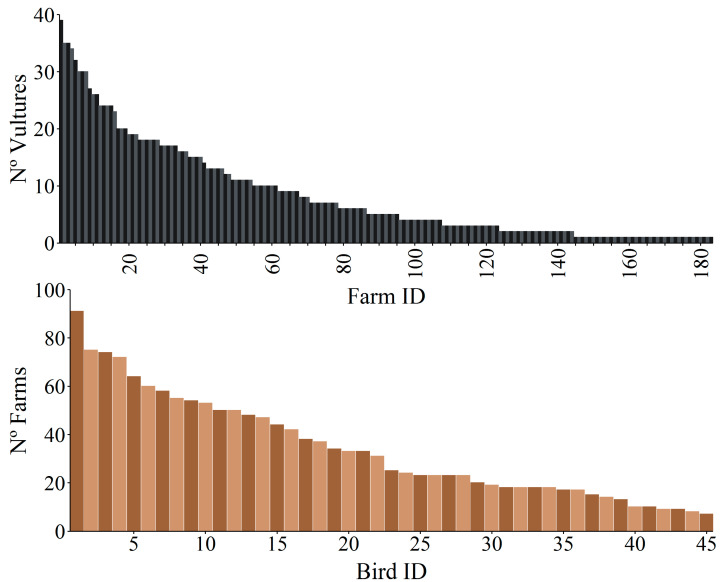
Frequency distribution of (**top**) the number of vultures that visited each farm (*Farm ID*) during the whole study period and (**bottom**) the number of farms that were visited by each tagged vulture (*Bird ID*) during the whole study period. There were 77 farms not visited by vultures that were not included in the figure.

The mean daily distance ± SD between all vultures’ locations selected as visits to farms and the nearest farm was 88.0 ± 41.1 m (*n* = 18,999) (see details and values per sex and territorial status in Figure S3). Overall, GPS-tagged birds visited at some point 70% of farms on Fuerteventura, with a maximum of 39 different vultures located in a single farm (Figure 3). Along the study period, vultures visited a mean of 34 ± 22 SD farms (range 7–91) (Figure 3). This high mean value was likely due to the higher use of farms by non-territorial males (Figure S2).

### 3.2. Model Results

**Figure 4:**
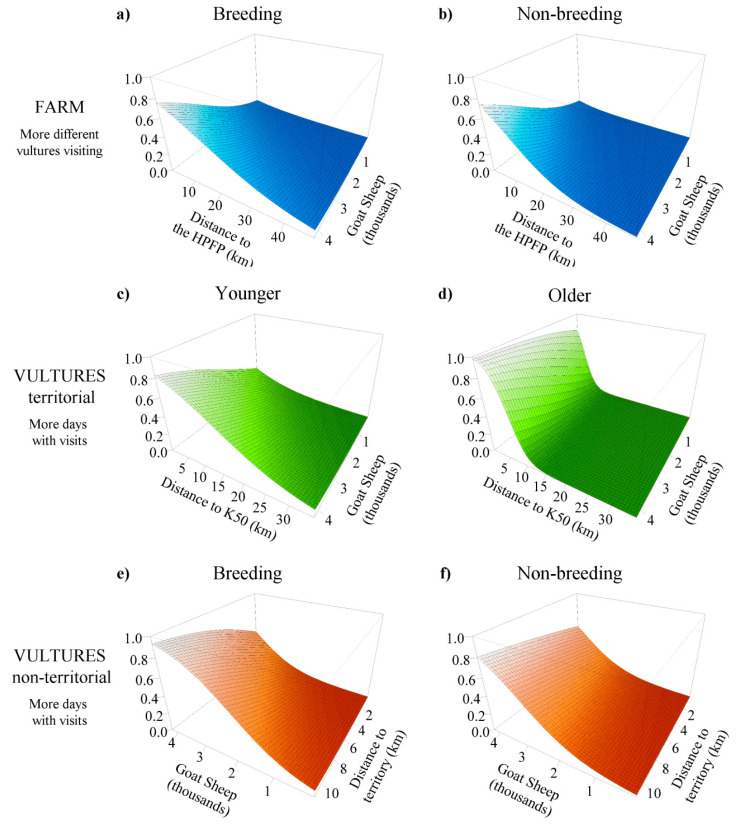
Effects of interacting variables in top-ranked models analyzing visits to farms by Egyptian Vultures. Effects of farm size (number of goats or sheep) on the probability of response variables: on top, *FARM* variable according to the distance to the nearest highly predictable feeding place (*Dist HPFP*) and season (**a**) breeding vs. (**b**) non-breeding, in the middle, *VULTURE territorial* variable regarding the distance to the nearest occupied territory (*Dist Terr*) and the (**c**) minimum (younger) or (**d**) maximum (older) age range; on the bottom, *VULTURE non-territorial* variable regarding the distance to the nearest core area of each individual (*Dist K50*) and season (**e**) breeding vs. (**f**) non-breeding. Values for explanatory variables in top-ranked models used in predictions are the mean of the range with some exceptions to clarify the effects: for *VULTURE territorial*, *Area K95* is the minimum of the range, *Dist Terr* is the maximun, *Sex* is male, and *Breeding* is breeding season; for *VULTURE non-territorial, Dist K50* is the minimum of the range.

Model selection for the *FARM* approach resulted in a single top-ranked model, describing the number of different vultures visiting a farm (Table S8) with a conditional R^2^ = 59.2 and a marginal R^2^ = 17.1 [58,59]. This model indicated that more vultures visited those farms that (1) had greater numbers of goats and sheep, (2) had greater probability of carcass abandonment, and (3) were further from roads (Table 4). Additionally, the interaction between breeding season and distance from the farm to the nearest highly predictable feeding place (HPFP) indicated that more vultures visited those farms (4) located closer to HPFP, with this effect being more pronounced during the non-breeding season (Table 4, Figure 4a,b).

The modeling procedure for the *VULTURE* approach focusing on territorial birds identified fifteen top-ranked models (Table S9) with a conditional R^2^ between 74.3 and 75, and a marginal R^2^ between 15.7 and 19.9. Model averaging (Table 4) revealed that territorial individuals visited the same farm on more days when the bird was (1) male and (2) had a smaller home range (determined by KernelUD 95%). In addition, the number of days increased (3) during the breeding season and also when the farm was (4) further from other breeding territories, (5) further from HPFP, (6) further from roads, and (7) had more livestock heads, and (8) a higher probability of carcass abandonment. There was (9) a negative effect of the distance to core areas that interacted with individual age. This indicates that the frequency of visits decreased monotonically with distance, but for older birds, activity was more focused on nearby farms, abruptly decreasing beyond 10 km from their core areas (Figure 4c,d).

Finally, four top-ranked models for non-territorial individuals received overwhelming support (Table S10) with a conditional R^2^ between 70.8 and 71.2, and a marginal R^2^ between 25.0 and 26.2. Model averaging (Table 4) showed that non-territorial individuals visited the same farm on more days when the bird (1) was older, (2) had a smaller home range, and when the farm was (3) further from roads, (4) closer to the individual’s core areas, (5) had more livestock heads, and (6) had a higher probability of carcass abandonment. There was also (7) a positive effect of the distance to breeding territories, which was more pronounced during the breeding season (Table 4 and Figure 4e,f).

Spatial autocorrelation in model residuals was low based on spline correlograms and Moran’s I between the 15 closest neighboring farms (Figures S4–S6). There was no overdispersion in *FARM* models, but *VULTURE* models showed a value of 2.7 and 2.2 for territorial and non-territorial, respectively. *VULTURE* models were additionally tested using a betabinomial distribution, as suggested for binomial overdispersed models [60]. Since results showed similar patterns to those obtained with binomial models (Tables S11 and S12), we decided to keep the binomial approach for simplicity and to facilitate comparison of results between studies.

## 4. Discussion

The management of scavenger bird populations through the provision of livestock carcasses has often been confronted with current legislation and health regulations. Sound scientific criteria should be used to solve this conflict and guide precise conservation actions. This is the first study to investigate the role of individual, spatial, and temporal factors determining the use of livestock farms by vultures. This was achieved thanks to the unprecedented information yielded by 45 GPS-tagged individuals from a population monitored for over 20 years and with a well-known social structure and foraging habits [13]. We found that the use of farms by vultures was not only determined by the availability of trophic resources and seasonality, but it also depended on individual vulture characteristics such as age and reproductive status. Additionally, the spatial distribution of resources in relation to the location of activity centers, such as territories and highly predictable feeding places, notably affected the use of livestock exploitations in a season-dependent way. Accordingly, for management planning, we must consider not only frequency and amount of food, but also the differences in the behavior of vultures and the distribution of their activity centers.

As expected, due to their greater food supply and lower disturbance, large-sized farms far from roads attracted more individuals and were more frequently visited by both territorial and non-territorial vultures. This pattern also had an important seasonal component, with farms being more visited during the breeding season. It is remarkable that differences between seasons arose despite our classification of breeding behavior into periods of six months, which does not consider potential differences within the breeding season (e.g., courting, hatching, chicks’ growth). During the breeding period, territorial individuals are involved in central place tasks (e.g., territory defense, nest attendance), which would lead to spatiotemporal foraging constraints. Although food predictability is lower at farms than at feeding stations and garbage dumps, food at farms is more widely distributed and likely more available in the surroundings of vultures’ territories. In contrast, non-territorial individuals are not attached to nesting sites, but the increasing frequency of visits to farms by breeding individuals could promote conspecific attraction. Additionally, goat and sheep births are typically concentrated in spring months, leading to higher amounts of livestock remains (e.g., remains of placentas and dead newborns) close to farms. Nevertheless, irrespective of their territorial status, vultures with smaller home ranges visited each farm more days, suggesting that a small number of farms may supply most of their feeding requirements.

Interestingly, the effect of vulture age on the number of visits to farms was different for territorial and non-territorial birds, and it was also affected by the distance from farms to core areas in the case of territorial individuals. Older non-territorial vultures would have better foraging skills and environmental knowledge than younger, less experienced birds, and they may preferentially visit certain well-known farms in a non-random way to increase foraging efficiency. However, territorial birds are attached to their territories. The fact that older territorial individuals rely more on those farms located closer to their core areas suggests not only that their better skills and knowledge allow them to optimize foraging efficiency at these farms, but also that they could obtain carrion from natural prey in the surroundings of their territories, without the need to visit more distant areas.

Sex-specific patterns also emerged within territorial vultures. Previous research in our study population showed that females (the larger sex) rely more on supplementary feeding stations than males, who spent less time at these places where competition for food may be high and where females show a dominant status [13,18]. As predicted, males were more likely to visit a farm than females, despite their similar roles in breeding tasks [61]. Although farms are considered a semi-predictable resource, they are probably unpredictable enough to avoid strong competition and displacement by highly dominant individuals. Moreover, this result reinforces the previously suggested idea of resource partitioning linked to sex-specific foraging strategies (13), which is a common phenomenon reported in sexually dimorphic birds [62,63].

Another important finding is the role of the spatial distribution of food and conspecifics in determining the use of farms by vultures. Firstly, both territorial and non-territorial birds avoid visiting those farms located close to breeding territories held by other individuals. Although the Egyptian Vulture is a relatively non-aggressive raptor, it actively defends its nesting area [64], including carcasses found in the close surroundings. In support of this, for non-territorial individuals, we found that farms in the proximity of territories were visited even more rarely during the breeding season, which was probably because of the higher territory attachment and investment in defense by territory holders during this period. Secondly, there was an important effect of the spatial location of highly predictable feeding places on the number of vultures visiting the farms. Overall, we observed that these food-rich sites attract birds from very distant areas [13,18], which can congregate in large numbers, especially outside the breeding season (up to 147 birds in a single day, [65]). This suggests that the attractiveness of these HPFP is not only due to the spatiotemporal predictability of food but also the opportunities they offer for socialization [65]. Consequently, those farms located near HPFP would also have high probabilities of being visited by more birds simply because of the large number of vultures that congregate [18]. Contrary to this idea, we found an apparent negative effect of the proximity of HPFP to farms on territorial individuals. It could be argued that this is due to the fact that half of the studied territories were more than 10 km away from HPFP and hence, territory attachment maintains birds’ activity further away from HPFP (Figure S7). Alternatively, this could also suggest that vultures breeding closer to the HPFP would preferentially forage at these highly predictable places instead of farms.

Overall, our results reveal general patterns of vulture visits to farms aimed at finding food. However, we lack detailed information of whether they obtain food on each visit to farms, and specially, the type and amount of food consumed. Further, Egyptian Vultures usually feed on small carrion scattered throughout more natural areas, but our approach does not allow assessing its relative importance and how it may influence the use of farms. Future studies should address these questions to fully understand the role of human-derived food sources in individual vultures’ foraging behavior and performance.

### Management Implications

There is consensus that allowing farmers to deposit livestock remains near their farms rather than concentrate food supplies at a few vulture restaurants may be beneficial for conservation purposes [2]. However, very little is known about the use of these scattered and semi-predictable food sites by vultures. Our findings can be useful to establish science-based guidelines for the management of carcasses in livestock farms. In addition to the amount and type of food, the design of targeted management planning should consider differences in individual behavior and the spatial distribution of key areas, such as breeding territories and highly predictable feeding places (HPFP).

Our results highlight the need to address conservation issues at different scales. On the one hand, and from a population point of view, the focus should be on larger farms providing more resources, especially those located in areas with low human disturbance. This not only increases the probability of visits because of birds’ mistrust of humans and their activities [66,67] but also reduces the probability of accidents with infrastructures [68]. The location of farms with respect to HPFP should also be considered, given its attractive effect on vultures, especially during the non-breeding season. At a smaller scale, the approach must be different. After recruiting, adult Egyptian Vultures exploit relatively small areas around the nesting sites (mean = 135.3 km^2^ ± 75.2 SD from 27 individuals and 35 breeding seasons, authors unpublished data). Our results show that in these areas, vultures still rely on large farms, but they territoriality imposes decisive constraints. This indicates that broad criteria based on the total area occupied by the species, such as creating a supplementary feeding station at the center of the distribution range, may fail to capture the requirements of the different fractions of the population (e.g., territorial breeders vs. non-territorial ones). Therefore, the spatial distribution of territories should be incorporated into the management of food resources. Moreover, Egyptian Vultures seem to rely more on farms as a reliable source of food as they acquire experience, so the management of livestock at farms could help ensure this food supply for territorial birds, while improving food availability in new areas for future recruits. Altogether, our results add to the growing knowledge on the complex ecological effects of predictable feeding places on vertebrate populations and communities [2,12,18].

## 5. Conclusions

The movement ecology approach used here allowed us to unravel the ecological drivers affecting the foraging behavior of an endangered vulture population, with clear conservation applications. Individual-based movements allowed the determination of patterns of use of a key and highly debated food resource for avian scavengers. By taking into account the complex network between spatial and temporal availability of food resources, characteristics of the livestock farms, individual bird traits, and constraints linked to seasonal behaviors, conservation measures aimed at managing semi-predictable resources found in farms will be successful for all fractions of the target population.

## Figures and Tables

**Table 1:** Predictions for each explanatory variable included in the analyses aimed at determining drivers of the selection of farms by Canarian Egyptian Vultures in Fuerteventura (Canary Islands). See Table 2 for a full description of considered variables.

Drivers	Variable	Predictions		References
		Territorial	Non-Territorial
Individual characteristics	Sex	Males will forage more than females on farms, because females heavily rely on supplementary feeding stations.		[13,18]
	Age	The probability of visiting a farm will be affected by individuals’ age due to differences in foraging strategies, skills, environmental knowledge, and movement patterns.		[13,19,20,21]
	Success	The probability of visiting a farm increases with successful breeding because of increased food demands.	-	
	AreaK95	The probability of visiting a farm increases with smaller home ranges, as the birds would concentrate searching effort in fewer and better-known areas.		
Main food resources provided by farms	Goats Sheep	Farms with more resources (number of heads of livestock) would receive more visits.		
	Carcass	Farms with more probability of abandoning livestock carcasses would receive more visits.		
Temporal constraints	Breeding	Farms will receive fewer visits during the breeding season due to spatial constraints derived from territory attendance (territorial birds) and prospecting behavior (non-territorial birds).		[18]
Spatial constraints relative to farms	Dist Terr	Visits increase with distance to the nearest occupied territory because owners defend trophic resources in areas close to their nests.		[18]
	Dist Road	Visits increase with distance to roads and urban areas because of less human disturbance.		[22]
	Dist Urb			
	Dist Nest	Visits decrease with distance to the nest because of central-place foraging restraints.	-	[4]
	Dist K50	Visits decrease with distance to core areas because of central-place foraging restraints.		[18]
	Dist HPFP	Visits decrease with distance to highly predictable feeding places (HPFP) because these places offer high amounts of food resources also acting as social meeting points. Therefore, individuals concentrate their activity around these points.		[13,23]

**Table 2:** Explanatory variables used to determine drivers of the selection of farms by Canarian Egyptian Vultures.

Variable	Description
**Fixed Factors**	
Dist HPFP ^a,b,c^	For each farm, distance from the centroid to the nearest HPFP (highly predictable feeding place): dump or supplementary feeding stations.
Dist Urb ^a,b,c^	For each farm, distance from the centroid to the nearest urban area.
Dist Road ^a,b,c^	For each farm, distance from the centroid to the nearest road.
Dist Terr ^b,c^	For each farm, individual, and semester, distance from the centroid of the farm to the nearest nest (different from their own nest in the case of territorial).
Dist K50 ^b,c^	For each farm, individual, and semester, distance from the centroid of the farm to the nearest core area defined by the KernelUD 50%.
Goat Sheep ^a,b,c^	For each farm and semester, the number of goats and sheep on the basis of livestock censuses (see Methods).
Carcass ^a,b,c^	For each farm, answer to the question about whether they abandon or not carcasses (affirmative answer = 1, negative = 0). When no information was available, it was based on the probability resulting from a predictive model performed (see Methods 2.4.1).
AreaK95 ^a,b,c^	For each individual and semester, the size of the area defined by KernelUD 95% (km^2^).
Breeding ^a,b,c^	For each semester, breeding season (1) or not (0).
Sex ^b,c^	For each individual, male (M) or female (F).
Age ^b,c^	For each individual and semester, age measured in years, calculated as the year of the semester minus year of birth.
Dist Nest ^b^	For each farm, breeding individual, and semester, distance from the centroid of the farm to the nest.
Success ^b^	For each individual and year, having at least one fledgling (1) or none (0) during the breeding season of the year.
**Random Factors**	
Semester ID ^a,b,c^	Period of six months defined as 1st semester or 2nd semester for each year from 2nd semester of 2013 to 2nd semester of 2016.
Farm ID ^a,b,c^	Farm identity, defined by one or more farms and the area around them with a threshold distance of 180 meters.
Bird ID ^b,c^	Individual identifier of each vulture.

^a^ Used in farm models; ^b^ Used in breeding vulture models; ^c^ Used in non-breeding vulture models.

**Table 3:** Results from Mann–Whitney U-tests assessing differences between sexes and breeding seasons in the number of farms included in Egyptian Vultures’ home ranges (N.farms HR) and in the number of farms visited by vultures (N.farms visited) accounting for their territorial status. Significant differences are bolded.

		**N.farms HR**			
		Territorial		Non-territ.	
		Breeding	Non-Breed.	Breeding	Non-Breed.
Differences between sexes	W	123.5	170	57	210
	*p*	0.665	1.000	**<0.01**	**<0.01**
		**N.farms Visited**			
		Territorial		Non-territ.	
		Breeding	Non-Breed.	Breeding	Non-Breed.
Differences between sexes	W	111	136.5	104	217
	*p*	0.374	0.312	**0.004**	**<0.001**
		Male	Female	Male	Female
Differences between seasons	W	152	185	203	95.5
	*p*	0.574	0.657	**<0.001**	**<0.001**

**Table 4:** Results of the best model for the response variables *FARM* and the full coefficients of model average for *territorial VULTURES* and *non-territorial VULTURES*. The estimates shown are the original outcome. See Table 1 for a full description of explanatory variables; 85% confidence intervals of the estimates are shown (7.5% and 92.5% limits). The reference level for factor Breeding is non-breeding season and for factor Sex is ‘Female’.

Variable	Estimate	Std. Error	7.5%	92.5%	RI
*FARM*					
(Intercept)	−4.41	0.19	−4.68	−4.13	
Goat Sheep	0.44	0.07	0.35	0.54	
Carcass	0.56	0.14	0.36	0.75	
Dist Road	0.60	0.13	0.41	0.79	
Dist HPFP	−0.86	0.14	−1.06	−0.66	
Breeding	0.49	0.21	0.19	0.79	
Breeding:Dist HPFP	0.31	0.07	0.22	0.40	
*Territorial VULTURES*					
(Intercept)	−7.06	0.34	−7.54	−6.57	
Sex	0.43	0.35	0.24	0.98	0.70
AreaK95	−0.45	0.04	−0.51	−0.39	1.00
Breeding	0.04	0.13	0.03	0.56	0.15
Dist Terr	0.80	0.06	0.72	0.88	1.00
Dist HPFP	0.27	0.19	0.12	0.56	0.80
Dist Road	0.45	0.25	0.23	0.80	0.87
Goat Sheep	0.35	0.09	0.22	0.48	1.00
Carcass	0.01	0.06	0.06	0.70	0.02
Dist K50	−1.33	0.05	−1.39	−1.26	1.00
Age	−0.12	0.15	−0.33	0.09	1.00
Age:Dist K50	−0.79	0.05	−0.86	−0.71	1.00
*Non-territorial VULTURES*					
(Intercept)	−7.13	0.21	−7.43	−6.82	
Age	0.03	0.04	0.00	0.12	0.51
AreaK95	−0.57	0.03	−0.61	−0.53	1.00
Dist Road	1.24	0.15	1.02	1.45	1.00
Dist K50	−0.68	0.02	−0.71	−0.65	1.00
Goat Sheep	0.66	0.04	0.61	0.71	1.00
Carcass	0.17	0.19	0.04	0.53	0.62
Dist Terr	0.21	0.05	0.14	0.28	1.00
Breeding	0.14	0.08	0.02	0.25	1.00
Breeding:Dist Terr	0.28	0.02	0.25	0.31	1.00

## Supplementary Materials

The following are available online at https://www.mdpi.com/2076-2615/10/11/2127/s1, Table S1: Number of GPS locations per semester and vulture used to calculate response variables *FARMS* and *VULTURES* for models assessing the drivers of the selection of farms by Canarian Egyptian Vultures, Table S2: Modeling the importance of random factors for models with the response variable *FARMS*, Table S3: Modeling the importance of random factors for models with the response variable *non-territorial VULTURES*, Table S4: Modeling the importance of random factors for models with the response variable *territorial VULTURES*, Table S5: Variance inflation factors (VIF) for explanatory variables in the datasets used for modeling the drivers of Egyptian Vulture visits to livestock farms in Fuerteventura (Canary Island), Table S6: Models without uninformative parameters predicting the probability of carcass abandonment in each farm in Fuerteventura (Canary Island), Table S7: Estimates and standard errors from the best model (lowest AICc) for variable *Carcass*., Table S8: Top-ranked models assessing drivers of the selection of farms by Canarian Egyptian Vultures for the response variable *FARMS*, Table S9: Top-ranked models assessing drivers of the selection of farms by Canarian Egyptian Vultures for the response variable *territorial VULTURES*, Table S10: Top-ranked models assessing drivers of the selection of farms by Canarian Egyptian Vultures for the response variable *non-territorial VULTURES*, Table S11: Top-ranked models assessing drivers of the selection of farms by Canarian Egyptian Vultures for the response variable *territorial VULTURES* using a betabinomial distribution to deal with overdispersion, Table S12: Top-ranked models assessing drivers of the selection of farms by Canarian Egyptian Vultures for the response variable *non-territorial VULTURES* using a betabinomial distribution to deal with overdispersion, Figure S1: Percentage of the study period per each GPS-tagged Egyptian Vulture with locations collected at different time intervals (i.e., time between two consecutive locations), Figure S2: Box-plots showing the differential use of farms by Egyptian Vultures according to sex, territorial status and breeding season, Figure S3: Frequency distribution of distance (m) from GPS-locations to farms considering (a) all locations used to analyze the use of farms by vultures; the daily mean calculated by vulture and farm for (b) all the individuals, (c) territorial females, (d) non-territorial females, (e) territorial males and (f) non-territorial males, Figure S4: Spline correlograms and values of Moran’s I for top-ranked model of the response variable *FARMS*, Figure S5: Spline correlograms and values of Moran’s I for top-ranked models of the response variable *non-territorial VULTURES*, Figure S6: Spline correlograms and values of Moran’s I for top-ranked models of the response variable *territorial VULTURES*, Figure S7: Boxplot showing distance between the largest core area defined by Kernel UD 50% for each semester and Egyptian Vulture and the closest highly predictable feeding place (HPFP), according to sex and breeding season.

## References

[1] Botha A.J., Andevski J., Bowden C.G., Gudka M., Safford R.J., Tavares J., Williams N.P. (2017). Multi-Species Action Plan to Conserve African-Eurasian Vultures (Vulture MsAP).

[2] Arrondo E., Cortés-Avizanda A., Donázar J.A. (2015). Temporally unpredictable supplementary feeding may benefit endangered scavengers. Ibis.

[3] Cortés-Avizanda A., Jovani R., Carrete M., Donázar J.A. (2012). Resource unpredictability promotes species diversity and coexistence in an avian scavenger guild: A field experiment. Ecology.

[4] Fluhr J., Benhamou S., Riotte-Lambert L., Duriez O. (2017). Assessing the risk for an obligate scavenger to be dependent on predictable feeding sources. Biol. Conserv..

[5] Moleón M., Sánchez-Zapata J.A., Margalida A., Carrete M., Owen-Smith N., Donázar J.A. (2014). Humans and scavengers: The evolution of interactions and ecosystem services. BioScience.

[6] Morales-Reyes Z., Pérez-García J.M., Moleón M., Botella F., Carrete M., Lazcano C., Moreno-Opo R., Margalida A., Donázar J.A., Sánchez-Zapata J.A. (2015). Supplanting ecosystem services provided by scavengers raises greenhouse gas emissions. Sci. Rep..

[7] Donázar J.A., Margalida A., Campión D. (2009). Vultures, Feeding Stations and Sanitary Legislation: A Conflict and Its Consequences from the Perspective of Conservation Biology.

[8] Margalida A., Carrete M., Sánchez-Zapata J.A., Donázar J.A. (2012). Good News for European Vultures. Science.

[9] Arrondo E., Moleón M., Cortés-Avizanda A., Jiménez J., Beja P., Sánchez-Zapata J.A., Donázar J.A. (2018). Invisible barriers: Differential sanitary regulations constrain vulture movements across country borders. Biol. Conserv..

[10] Cortés-Avizanda A., Carrete M., Donázar J.A. (2010). Managing supplementary feeding for avian scavengers: Guidelines for optimal design using ecological criteria. Biol. Conserv..

[11] Moreno-Opo R., Trujillano A., Arredondo Á., González L.M., Margalida A. (2015). Manipulating size, amount and appearance of food inputs to optimize supplementary feeding programs for European vultures. Biol. Conserv..

[12] Cortés-Avizanda A., Blanco G., DeVault T.L., Markandya A., Virani M.Z., Brandt J., Donázar J.A. (2016). Supplementary feeding and endangered avian scavengers: Benefits, caveats, and controversies. Front. Ecol. Environ..

[13] van Overveld T., García-Alfonso M., Dingemanse N.J., Bouten W., Gangoso L., de la Riva M., Serrano D., Donázar J.A. (2018). Food predictability and social status drive individual resource specializations in a territorial vulture. Sci. Rep..

[14] Medina F.M. (1999). Alimentación del alimoche, *Neophron percnopterus* (L.), en Fuerteventura, Islas Canarias (Aves, Accipitridae). Vieraea.

[15] Gangoso L., Donázar J.A., Scholz S., Palacios C.J., Hiraldo F. (2006). Contradiction in conservation of island ecosystems: Plants, introduced herbivores and avian scavengers in the Canary Islands. Biodivers. Conserv..

[16] Blanco G., Cortés-Avizanda A., Frías Ó., Arrondo E., Donázar J.A. (2019). Livestock farming practices modulate vulture diet-disease interactions. Glob. Ecol. Conserv..

[17] García-Alfonso M., Morales-Reyes Z., Gangoso L., Bouten W., Sánchez-Zapata J.A., Serrano D., Donázar J.A. (2018). Probing into farmers’ perceptions of a globally endangered ecosystem service provider. Ambio.

[18] García-Heras M.-S., Cortés-Avizanda A., Donázar J.A. (2013). Who are we feeding? Asymmetric individual use of surplus food resources in an insular population of the endangered Egyptian Vulture *Neophron percnopterus*. PLoS ONE.

[19] Sanz-Aguilar A., Jovani R., Melián C.J., Pradel R., Tella J.L. (2015). Multi-event capture-recapture analysis reveals individual foraging specialization in a generalist species. Ecology.

[20] Krüger S., Reid T., Amar A. (2014). Differential Range Use between Age Classes of Southern African Bearded Vultures *Gypaetus barbatus*. PLoS ONE.

[21] Wolfson D.W., Fieberg J.R., Andersen D.E. (2020). Juvenile Sandhill Cranes exhibit wider ranging and more exploratory movements than adults during the breeding season. Ibis.

[22] Gavashelishvili A., McGrady M.J. (2006). Geographic information system-based modelling of vulture response to carcass appearance in the Caucasus. J. Zool..

[23] Monsarrat S., Benhamou S., Sarrazin F., Bessa-Gomes C., Bouten W., Duriez O. (2013). How Predictability of Feeding Patches Affects Home Range and Foraging Habitat Selection in Avian Social Scavengers?. PLoS ONE.

[24] Rodríguez Delgado O., García Gallo A., Reyes Betancort J.A. (2000). Estudio fitosociológico de la vegetación actual de Fuerteventura (Islas Canarias). Vieraea.

[25] García-Martínez A., Olaizola A., Bernués A. (2009). Trajectories of evolution and drivers of change in European mountain cattle farming systems. Animal.

[26] Canarian Government Estadistica Ganadera. http://www.gobiernodecanarias.org/istac/.

[27] Donázar J.A., Palacios C.J., Gangoso L., Ceballos O., Gonzáles M.J., Hiraldo F. (2002). Conservation status and limiting factors in the endangered population of Egyptian Vulture (*Neophron percnopterus*) in the Canary Islands. Biol. Conserv..

[28] Agudo R., Carrete M., Alcaide M., Rico C., Hiraldo F., Donázar J.A. (2012). Genetic diversity at neutral and adaptive loci determines individual fitness in a long-lived territorial bird. Proc. Biol. Sci..

[29] Donázar J.A., Negro J.J., Palacios C.J., Gangoso L., Godoy J.A., Ceballos O., Hiraldo F., Capote N. (2002). Description of a new subspecies of the Egyptian Vulture (Accipitridae: *Neophron percnopterus*) from the Canary Islands. J. Raptor Res..

[30] Donázar J.A., Barbosa J.M., García-Alfonso M., van Overveld T., Gangoso L., de la Riva M. (2020). Too much is bad: Increasing numbers of livestock and conspecifics reduce body mass in an avian scavenger. Ecol. Appl..

[31] Badia-Boher J.A., Sanz-Aguilar A., de la Riva M., Gangoso L., van Overveld T., García-Alfonso M., Luzardo O.P., Suarez-Pérez A., Donázar J.A. (2019). Evaluating European LIFE conservation projects: Improvements in survival of an endangered vulture. J. Appl. Ecol..

[32] Bouten W., Baaij E.W., Shamoun-Baranes J., Camphuysen K.C.J. (2013). A flexible GPS tracking system for studying bird behaviour at multiple scales. J. Ornithol..

[33] Sergio F., Tavecchia G., Tanferna A., López Jiménez L., Blas J., De Stephanis R., Marchant T.A., Kumar N., Hiraldo F. (2015). No effect of satellite tagging on survival, recruitment, longevity, productivity and social dominance of a raptor, and the provisioning and condition of its offspring. J. Appl. Ecol..

[34] MAGRAMA Spanish Ministry of Agriculture, Food and Environment. http://www.magrama.gog.es.

[35] Centro Nacional de Información Geofráfica [CNIG] Modelo Digital de Elevaciones 25. http://centrodedescargas.cnig.es/CentroDescargas/catalogo.do?Serie=MAPLI.

[36] Klaassen R.H.G., Schlaich A.E., Bouten W., Koks B.J. (2017). Migrating Montagu’s harriers frequently interrupt daily flights in both Europe and Africa. J. Avian Biol..

[37] Zuur A.F., Ieno E.N., Walker N.J., Savelieve A.A., Smith G.M., Gails M., Krickeberg K., Samet J.M., Tsiatis A., Wong W. (2009). Mixed Effects Models and Extensions in Ecology with R.

[38] Sugiura N. (1978). Further analysis of the data by akaike’ s information criterion and the finite corrections. Commun. Stat. Theory Methods.

[39] Bolker B.M., Brooks M.E., Clark C.J., Geange S.W., Poulsen J.R., Stevens M.H.H., White J.S.S. (2009). Generalized linear mixed models: A practical guide for ecology and evolution. Trends Ecol. Evol..

[40] Bolker B.M. GLMM FAQ. https://bbolker.github.io/mixedmodels-misc/glmmFAQ.html.

[41] Gelman A., Hill J. (2007). Linear regression: Before and after fitting the model. Data Analysis Using Regression and Multilevel/Hierarchical Models.

[42] Graham M.H. (2003). Statistical confronting multicollinearity in ecological. Ecology.

[43] Arnold T.W. (2010). Uninformative parameters and model selection using Akaike’s information criterion. J. Wildl. Manag..

[44] Burnham K.P., Anderson D.R. (2002). Model Selection and Multimodel Inference: A Practical Information-Theoretic Approach.

[45] Crawley M.J. (2002). Statistical Computing: An Introduction to Data Analysis Using S-Plus.

[46] Nakagawa S., Schielzeth H. (2013). A general and simple method for obtaining R^2^ from generalized linear mixed-effects models. Methods Ecol. Evol..

[47] Bjørnstad O.N., Falck W. (2001). Nonparametric spatial covariance functions: Estimation and testing. Environ. Ecol. Stat..

[48] Moreira F., Martins R.C., Catry I., D’Amico M. (2018). Drivers of power line use by white storks: A case study of birds nesting on anthropogenic structures. J. Appl. Ecol..

[49] R Core Team R (2017). A Language and Environment For Statistical Computing.

[50] Bates D., Maechler M., Bolker B., Walker S. (2015). Fitting Linear Mixed-Effects Models Using lme4. J. Stat. Softw..

[51] Brooks M.E., Kristensen K., van Benthem K.J., Magnusson A., Berg C.W., Nielsen A., Skaug H.J., Mächler M., Bolker B.M. (2017). glmmTMB balances speed and flexibility among packages for zero-inflated generalized linear mixed modeling. R J..

[52] Mazerolle M.J. (2016). AICcmodavg: Model Selection and Multimodel Inference Based on (Q)AIC(c). R Package, version 2.0-4. https://cran.r-project.org/package=AICcmodavg.

[53] Barton K. (2016). MuMIn: Multi-Model Inference. R Package, version 1.15.6. https://CRAN.R-project.org/package=MuMIn.

[54] Naimi B., Hamm N.A.S., Groen T.A., Skidmore A.K., Toxopeus A.G. (2014). Where is positional uncertainty a problem for species distribution modelling?. Ecography.

[55] Barbosa A.M., Brown J.A., Jimenez-Valverde A., Real R. (2016). modEvA: Model Evaluation and Analysis. R Package, version 1.3.2. https://CRAN.R-project.org/package=modEvA.

[56] Hervé M. (2018). RVAideMemoire: Testing and Plotting Procedures for Biostatistics. R Package, version 0.9-69-3. https://CRAN.R-project.org/package=RVAideMemoire.

[57] Guisan A., Zimmermann N.E. (2000). Predictive habitat distribution models in ecology. Ecol. Modell..

[58] Nakagawa S., Johnson P.C.D., Schielzeth H. (2017). The coefficient of determination R^2^ and intra-class correlation coefficient from generalized linear mixed-effects models revisited and expanded. J. R. Soc. Interface.

[59] Barton K. (2019). MuMIn: Multi-Model Inference. R Package, version 1.43.6. https://CRAN.R-project.org/package=MuMIn.

[60] Harrison X.A. (2015). A comparison of observation-level randomeffect and Beta-Binomial models for modelling overdispersion in Binomial data in ecology & evolution. PeerJ.

[61] Morant Etxebarria J., López-López P., Zuberogoitia Arroyo I. (2019). Parental investment asymmetries of a globally endangered scavenger: Unravelling the role of gender, weather conditions and stage of the nesting cycle. Bird Study.

[62] Newton I. (1979). Population Ecology of Raptors.

[63] Shaffer S.A., Weimerskirch H., Costa D.P. (2001). Functional significance of sexual dimorphism in Wandering Albatrosses, *Diomedea exulans*. Funct. Ecol..

[64] Ceballos O., Donázar J.A. (1988). Actividad, uso del espacio y cuidado parental en una pareja de alimoches (*Neophron percnopterus*) durante el periodo de dependencia de los pollos. Ecología.

[65] Van Overveld T., Gangoso L., García-Alfonso M., Bouten W., de la Riva M., Donázar J.A. (2020). Seasonal grouping dynamics in a territorial vulture: Ecological drivers and social consequences. Behav. Ecol. Sociobiol..

[66] Donázar J.A., Ceballos O., Cortés-Avizanda A. (2018). Tourism in protected areas: Disentangling road and traffic effects on intra-guild scavenging processes. Sci. Total Environ..

[67] Bautista L.M., García J.T., Calmaestra R.G., Palacín C., Martín C.A., Morales M.B., Bonal R., Viñuela J. (2004). Effect of weekend road traffic on the use of space by raptors. Conserv. Biol..

[68] Tejera G., Rodríguez B., Armas C., Rodríguez A. (2018). Wildlife-vehicle collisions in Lanzarote biosphere reserve, Canary Islands. PLoS ONE.

