# Supplementary material for: Vultures and Livestock: The Where, When, and Why of Visits to Farms
Source: Animals (Basel). 2020 Nov 16;10(11):2127. doi: 10.3390/ani10112127 (PMC7698296; doi:10.3390/ani10112127)
Supplement: Supplementary file 1 [file animals-10-02127-s001.zip › supplementary 10_Table S11-12.pdf]

Table S11. Top-ranked models assessing drivers of the selection of farms by Canarian Egyptian Vultures for the response variable *territorial VULTURES* using a betabinomial distribution to deal with overdispersion. See Table 2 for a full description of explanatory variables. The number of parameters (K), the AICc, the difference in AICc between each model and the best model (ΔAICc), Akaike weights (wi) and the dispersion parameter are shown. "1|Bird ID", "1|Farm ID" and "1|Semester ID" indicate the random effect of bird, farm, and semester identity respectively. Only the 20 highest-ranked and the null (intercept plus random terms) models are shown. All combinations from only the intercept to all considered fixed effects were tested.

Model	Variable	K	AICc	ΔAICc	wi	Dispersion
mod1	Age + Age:Dist K50 + AreaK95 + Goat Sheep + Dist K50 + Dist HPFP + Dist Road + Dist Terr + Sex + (1|Farm ID) + (1|Bird ID) + (1|Semester ID)	14	4411.33	0.000	0.417	19.3
mod2	Age + Age:Dist K50 + AreaK95 + Breeding + Goat Sheep + Dist K50 + Dist HPFP + Dist Road + Dist Terr + Sex + (1|Farm ID) + (1|Bird ID) + (1|Semester ID)	15	4411.76	0.436	0.336	19.3
mod3	Age + Age:Dist K50 + AreaK95 + Goat Sheep + Dist K50 + Dist Road + Dist Terr + Sex + (1|Farm ID) + (1|Bird ID) + (1|Semester ID)	13	4414.25	2.924	0.097	19.3
mod4	Age + Age:Dist K50 + AreaK95 + Breeding + Goat Sheep + Dist K50 + Dist Road + Dist Terr + Sex + (1|Farm ID) + (1|Bird ID) + (1|Semester ID)	14	4414.63	3.299	0.08	19.3
mod5	Age + Age:Dist K50 + AreaK95 + Goat Sheep + Dist K50 + Dist HPFP + Dist Terr + Sex + (1|Farm ID) + (1|Bird ID) + (1|Semester ID)	13	4416.47	5.143	0.032	19.4
mod6	Age + Age:Dist K50 + AreaK95 + Goat Sheep + Dist K50 + Dist HPFP + Dist Road + Dist Terr + (1|Farm ID) + (1|Bird ID) + (1|Semester ID)	13	4418.45	7.119	0.012	19.5
mod7	Age + Age:Dist K50 + AreaK95 + Carcass + Goat Sheep + Dist K50 + Dist Terr + Sex + (1|Farm ID) + (1|Bird ID) + (1|Semester ID)	13	4419.69	8.358	0.006	19.3
mod8	Age + Age:Dist K50 + AreaK95 + Goat Sheep + Dist K50 + Dist Terr + Sex + (1|Farm ID) + (1|Bird ID) + (1|Semester ID)	12	4420.57	9.246	0.004	19.4
mod9	Age + Age:Dist K50 + AreaK95 + Breeding + Goat Sheep + Dist K50 + Dist Terr + Sex + (1|Farm ID) + (1|Bird ID) + (1|Semester ID)	13	4421.04	9.709	0.003	19.3
mod10	Age + Age:Dist K50 + AreaK95 + Goat Sheep + Dist K50 + Dist Road + Dist Terr + (1|Farm ID) + (1|Bird ID) + (1|Semester ID)	12	4421.33	9.999	0.003	19.5

Model	Variable	K	AICc	ΔAICc	wi	Dispersion
mod11	Age + Age:Dist K50 + AreaK95 + Breeding + Goat Sheep + Dist K50 + Dist Road + Dist Terr + (1|Farm ID) + (1|Bird ID) + (1|Semester ID)	13	4421.73	10.404	0.002	19.5
mod12	Age + Age:Dist K50 + AreaK95 + Dist K50 + Dist HPFP + Dist Road + Dist Terr + Sex + (1|Farm ID) + (1|Bird ID) + (1|Semester ID)	13	4422.81	11.485	0.001	19.5
mod13	Age + Age:Dist K50 + AreaK95 + Breeding + Dist K50 + Dist HPFP + Dist Road + Dist Terr + Sex + (1|Farm ID) + (1|Bird ID) + (1|Semester ID)	14	4422.85	11.518	0.001	19.5
mod14	AreaK95 + Breeding + Goat Sheep + Dist K50 + Dist HPFP + Dist Road + Dist Terr + Sex + Success + Success:Dist K50 + (1|Farm ID) + (1|Bird ID) + (1|Semester ID)	15	4423.09	11.764	0.001	17.8
mod15	Age + Age:Dist K50 + AreaK95 + Goat Sheep + Dist K50 + Dist HPFP + Dist Terr + (1|Farm ID) + (1|Bird ID) + (1|Semester ID)	12	4423.54	12.216	0.001	19.5
mod16	AreaK95 + Goat Sheep + Dist K50 + Dist HPFP + Dist Road + Dist Terr + Sex + Success + Success:Dist K50 + (1|Farm ID) + (1|Bird ID) + (1|Semester ID)	14	4424.01	12.676	0.001	17.9
mod17	Age + Age:Dist K50 + AreaK95 + Breeding + Dist K50 + Dist Road + Dist Terr + Sex + (1|Farm ID) + (1|Bird ID) + (1|Semester ID)	13	4425.68	14.355	0.000	19.5
mod18	Age + Age:Dist K50 + AreaK95 + Dist K50 + Dist Road + Dist Terr + Sex + (1|Farm ID) + (1|Bird ID) + (1|Semester ID)	12	4425.70	14.375	0.000	19.5
mod19	AreaK95 + Breeding + Goat Sheep + Dist K50 + Dist Road + Dist Terr + Sex + Success + Success:Dist K50 + (1|Farm ID) + (1|Bird ID) + (1|Semester ID)	14	4426.26	14.931	0.000	17.8
mod20	Age + Age:Dist K50 + AreaK95 + Carcass + Goat Sheep + Dist K50 + Dist Terr + (1|Farm ID) + (1|Bird ID) + (1|Semester ID)	12	4426.75	15.423	0.000	19.5
mod0	(1|Farm ID) + (1|Bird ID) + (1|Semester ID)	5	4687.59	276.266	0.000	9.8

Table S12. Top-ranked models assessing drivers of the selection of farms by Canarian Egyptian Vultures for the response variable *non-territorial VULTURES* using a betabinomial distribution to deal with overdispersion. See Table 2 for a full description of explanatory variables. The number of parameters (K), the AICc, the difference in AICc between each model and the best model (ΔAICc), Akaike weights (wi) and the dispersion parameter are shown. "1|Bird ID", "1|Farm ID" and "1|Semester ID" indicate the random effect of bird, farm, and semester identity respectively. Only the 20 highest-ranked and the null (intercept plus random terms) models are shown. All combinations from only the intercept to all considered fixed effects were tested.

Model	Variable	K	AICc	ΔAICc	wi	Dispersion
mod1	AreaK95 + Breeding + Breeding:Dist Terr + Carcass + Goat Sheep + Dist K50 + Dist Road + Dist Terr + (1|Farm ID) + (1|Bird ID) + (1|Semester ID)	13	13388.05	0.000	0.394	31.1
mod2	AreaK95 + Breeding + Breeding:Dist HPFP + Carcass + Goat Sheep + Dist K50 + Dist HPFP + Dist Road + Dist Terr + (1|Farm ID) + (1|Bird ID) + (1|Semester ID)	14	13388.64	0.597	0.292	30.9
mod3	AreaK95 + Breeding + Breeding:Dist Terr + Goat Sheep + Dist K50 + Dist Road + Dist Terr + (1|Farm ID) + (1|Bird ID) + (1|Semester ID)	12	13391.33	3.284	0.076	31.1
mod4	AreaK95 + Carcass + Goat Sheep + Goat Sheep:Dist Road + Dist K50 + Dist Road + Dist Terr + (1|Farm ID) + (1|Bird ID) + (1|Semester ID)	12	13391.67	3.619	0.064	30.8
mod5	AreaK95 + Breeding + Breeding:Dist HPFP + Goat Sheep + Dist K50 + Dist HPFP + Dist Road + Dist Terr + (1|Farm ID) + (1|Bird ID) + (1|Semester ID)	13	13391.95	3.902	0.056	31.0
mod6	AreaK95 + Breeding + Breeding:Dist HPFP + Carcass + Goat Sheep + Dist K50 + Dist HPFP + Dist Road + (1|Farm ID) + (1|Bird ID) + (1|Semester ID)	13	13393.13	5.086	0.031	30.9
mod7	AreaK95 + Carcass + Goat Sheep + Goat Sheep:Dist HPFP + Dist K50 + Dist HPFP + Dist Road + Dist Terr + (1|Farm ID) + (1|Bird ID) + (1|Semester ID)	13	13393.82	5.771	0.022	30.8
mod8	AreaK95 + Goat Sheep + Goat Sheep:Dist Road + Dist K50 + Dist Road + Dist Terr + (1|Farm ID) + (1|Bird ID) + (1|Semester ID)	11	13394.66	6.616	0.014	30.8
mod9	AreaK95 + Carcass + Goat Sheep + Goat Sheep:areaK95 + Dist K50 + Dist Road + Dist Terr + (1|Farm ID) + (1|Bird ID) + (1|Semester ID)	12	13396.39	8.343	0.006	30.9

Model	Variable	K	AICc	ΔAICc	wi	Dispersion
mod10	AreaK95 + Carcass + Goat Sheep + Goat Sheep:Dist Road + Dist K50 + Dist Road + (1|Farm ID) + (1|Bird ID) + (1|Semester ID)	11	13396.68	8.636	0.005	30.8
mod11	Age + Age:areaK95 + AreaK95 + Carcass + Goat Sheep + Dist K50 + Dist Road + Dist Terr + (1|Farm ID) + (1|Bird ID) + (1|Semester ID)	13	13397.03	8.983	0.004	30.7
mod12	Age + AreaK95 + Carcass + Goat Sheep + Dist K50 + Dist Road + Dist Terr + Sex + Sex:Age + (1|Farm ID) + (1|Bird ID) + (1|Semester ID)	14	13397.09	9.040	0.004	30.7
mod13	AreaK95 + Breeding + Breeding:Dist HPFP + Goat Sheep + Dist K50 + Dist HPFP + Dist Road + (1|Farm ID) + (1|Bird ID) + (1|Semester ID)	12	13397.27	9.224	0.004	30.9
mod14	AreaK95 + Carcass + Goat Sheep + Goat Sheep:Dist HPFP + Dist K50 + Dist HPFP + Dist Road + (1|Farm ID) + (1|Bird ID) + (1|Semester ID)	12	13397.98	9.929	0.003	30.7
mod15	AreaK95 + Goat Sheep + Goat Sheep:Dist HPFP + Dist K50 + Dist HPFP + Dist Road + Dist Terr + (1|Farm ID) + (1|Bird ID) + (1|Semester ID)	12	13398.14	10.090	0.003	30.8
mod16	AreaK95 + Carcass + Carcass:Dist Road + Goat Sheep + Dist K50 + Dist Road + Dist Terr + (1|Farm ID) + (1|Bird ID) + (1|Semester ID)	12	13398.25	10.208	0.002	30.6
mod17	Age + Age:Dist Terr + AreaK95 + Carcass + Goat Sheep + Dist K50 + Dist Road + Dist Terr + (1|Farm ID) + (1|Bird ID) + (1|Semester ID)	13	13398.70	10.657	0.002	30.8
mod18	AreaK95 + Carcass + Goat Sheep + Goat Sheep:Carcass + Dist K50 + Dist Road + Dist Terr + (1|Farm ID) + (1|Bird ID) + (1|Semester ID)	12	13399.27	11.223	0.001	30.7
mod19	AreaK95 + Carcass + Carcass:areaK95 + Goat Sheep + Dist K50 + Dist Road + Dist Terr + (1|Farm ID) + (1|Bird ID) + (1|Semester ID)	12	13399.30	11.256	0.001	30.7
mod20	AreaK95 + Carcass + Goat Sheep + Dist K50 + Dist Road + Dist Terr + Sex + Sex:Dist Road + (1|Farm ID) + (1|Bird ID) + (1|Semester ID)	13	13399.39	11.345	0.001	30.8
mod0	(1|Farm ID) + (1|Bird ID) + (1|Semester ID)	5	13835.46	447.414	0.000	23.0
