# Supplementary material for: Vultures and Livestock: The Where, When, and Why of Visits to Farms
Source: Animals (Basel). 2020 Nov 16;10(11):2127. doi: 10.3390/ani10112127 (PMC7698296; doi:10.3390/ani10112127)

Mann-Whitney tests for differences between territorial and non-territorial individuals in the distance from the largest core area (Kernel UD 50%) for each semester and Egyptian Vulture to the closest highly predictable feeding place (HPFP), according to sex and breeding season. For the breeding season, we found W = 30 (p-value < 0.001, N = 34) and W = 107 (p-value < 0.009, N = 42) between females and males respectively, whereas for the non-breeding season, we found W = 259 (p-value = 0.823, N = 47) and W = 179 (p-value < 0.009, N = 55). Values for distances in each case are shown in Figure S7.

Figure S7. Boxplot showing distance between the largest core area defined by Kernel UD 50% for each semester and Egyptian Vulture and the closest highly predictable feeding place (HPFP). Differences between sex, breeding seasons and territorial status are shown. The line within boxes indicates the median, the edges of the boxes the first (Q1) and third (Q3) quartiles, and the whiskers extend 1.5 times the interquartile range.

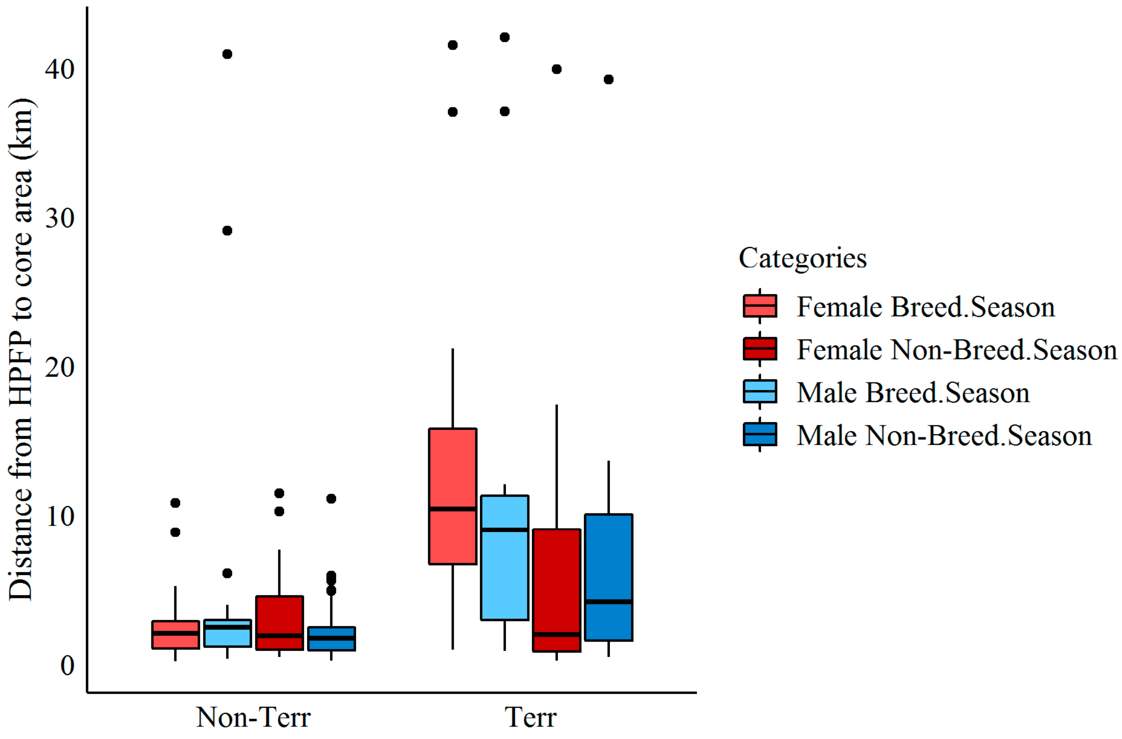

Supplement: Supplementary file 1 [file animals-10-02127-s001.zip › supplementary 11_Figure S7.pdf]
