# Supplementary material for: Vultures and Livestock: The Where, When, and Why of Visits to Farms
Source: Animals (Basel). 2020 Nov 16;10(11):2127. doi: 10.3390/ani10112127 (PMC7698296; doi:10.3390/ani10112127)
Supplement: Supplementary file 1 [file animals-10-02127-s001.zip › supplementary 1_Table S1.pdf]

**Table S1.** Number of GPS locations per semester and vulture used to calculate response variables *FARMS* and *VULTURES* for models assessing the drivers of the selection of farms by Canarian Egyptian Vultures. Bird identities, capture dates, sex (F: females, M: males) and year of birth are shown. The rest of column names indicate the year and the first month of each semester. Territorial individuals of each semester are marked with '*'.

| Bird ID | Capture date | Sex | Birth | 2013-07-01 | 2014-01-01 | 2014-07-01 | 2015-01-01 | 2015-07-01 | 2016-01-01 | 2016-07-01 |
|---|---|---|---|---|---|---|---|---|---|---|
| 225 | 2013-06-13 | F | 2009 | 182,593 | 307,169 | 31,087 | 36,958 | 33,731 | 35,407 | 35,315 |
| 226 | 2013-06-13 | F | 2009 | 212,466 | 224,884* | 44,981* | 62,671* | 33,900* | 35,250 | 35,808 |
| 228 | 2013-06-13 | F | 2009 | 336,114 | 514,973* | 273,900* | 76,302* | 33,242* | 0 | 0 |
| 231 | 2013-06-13 | M | 2010 | 0 | 244,194 | 37,089 | 42,808 | 34,009 | 34,857 | 35,369 |
| 237 | 2013-06-13 | M | 2010 | 137,946 | 257,772 | 66,045 | 53,381 | 36,651 | 36,750* | 65,445* |
| 2X1 | 2013-06-16 | M | 2010 | 289,060 | 231,572 | 158,249 | 81,002 | 35,541 | 29,176* | 0 |
| 2X2 | 2013-06-16 | F | 2010 | 243,943 | 392,062 | 249,147 | 105,896 | 36,099 | 29,355* | 140,345* |
| 2X4 | 2013-06-16 | M | 2010 | 137,289 | 126,758 | 62,112 | 44,783* | 34,805* | 0 | 0 |
| 2X5 | 2013-06-16 | M | 2010 | 242,187 | 558,107 | 254,886 | 73,938 | 36,504 | 36,506* | 154,243* |
| 22T | 2013-06-20 | M | 2010 | 155,156 | 252,937 | 150,946 | 83,312* | 36,856* | 32,446* | 36,705* |
| 3FC | 2013-06-20 | M | 2009 | 182,572 | 170,345* | 34,103* | 39,115* | 42,238* | 35,735* | 35,427* |
| 3FF | 2013-06-20 | F | 2010 | 145,374 | 65,195 | 45,048 | 0 | 35,726 | 34,310* | 32,495* |
| 22C | 2013-06-26 | M | 2009 | 232,399 | 261,332 | 42,833 | 59,319 | 36,634 | 36,563 | 174,189 |
| 22X | 2013-06-26 | M | 2010 | 237,707 | 286,786 | 37,511 | 101,312 | 36,536 | 36,837 | 37,081 |
| 2X3 | 2013-06-27 | F | 2010 | 13,060 | 215,137 | 93,738 | 96,148 | 46,217 | 89,250 | 135,623 |
| 3NP | 2013-10-23 | M | 2013 | 0 | 423,169 | 206,678 | 61,314 | 36,936 | 36,406 | 149,653 |
| 234 | 2014-05-28 | M | 2010 | 0 | 0 | 17,905 | 71,244 | 36,512 | 0 | 0 |
| 371 | 2014-05-28 | M | 2011 | 0 | 0 | 20,563 | 59,462 | 36,534 | 35,499* | 179,698* |
| 224 | 2014-05-31 | M | 2009 | 0 | 0 | 22,118 | 41,069* | 33,463* | 31,970* | 33,104* |
| 341 | 2014-05-31 | F | 2011 | 0 | 0 | 20,326 | 56,448 | 36,939 | 36,952 | 175,326 |
| 2XV | 2014-06-02 | F | 2011 | 0 | 0 | 10,838 | 20,622 | 31,737 | 32,006 | 30,730 |
| 2XM | 2015-05-23 | F | 2011 | 0 | 0 | 0 | 0 | 37,105 | 36,874 | 193,785 |

| Bird ID | Capture date | Sex | Birth | 2013-07-01 | 2014-01-01 | 2014-07-01 | 2015-01-01 | 2015-07-01 | 2016-01-01 | 2016-07-01 |
|---|---|---|---|---|---|---|---|---|---|---|
| 3PU | 2015-05-23 | M | 2010 | 0 | 0 | 0 | 0 | 32,657* | 31,113* | 30,598* |
| 3PX | 2015-05-23 | F | 2004 | 0 | 0 | 0 | 0 | 34,070* | 29,369* | 122,571* |
| 2XC | 2015-05-25 | F | 2006 | 0 | 0 | 0 | 0 | 36,672* | 32,737* | 126,258* |
| 3FA | 2015-05-25 | M | 2001 | 0 | 0 | 0 | 0 | 35,218* | 30,119* | 33,796* |
| 221 | 2015-09-16 | F | 2009 | 0 | 0 | 0 | 0 | 0 | 52,446* | 72,711* |
| 222 | 2015-09-16 | M | 2009 | 0 | 0 | 0 | 0 | 0 | 68,544* | 122,261* |
| 254 | 2015-09-16 | F | 2007 | 0 | 0 | 0 | 0 | 0 | 66,554* | 91,663* |
| 255 | 2015-09-16 | F | 2007 | 0 | 0 | 0 | 0 | 0 | 89,264* | 122,552* |
| 370 | 2015-09-16 | M | 2011 | 0 | 0 | 0 | 0 | 0 | 89,576 | 87,604 |
| 3AC | 2015-09-16 | M | 2012 | 0 | 0 | 0 | 0 | 0 | 84,682 | 80,361 |
| 3XV | 2015-09-16 | F | 2005 | 0 | 0 | 0 | 0 | 0 | 34,855* | 41,223* |
| 40C | 2015-09-18 | F | 2010 | 0 | 0 | 0 | 0 | 0 | 51,796* | 50,163* |
| 40H | 2015-09-18 | F | 2006 | 0 | 0 | 0 | 0 | 0 | 30,486* | 43,218* |
| 22P | 2015-09-19 | F | 2008 | 0 | 0 | 0 | 0 | 0 | 51,272* | 64,373* |
| 2XX | 2015-09-19 | M | 2011 | 0 | 0 | 0 | 0 | 0 | 83,193 | 87,989 |
| 20H | 2015-09-21 | M | 2006 | 0 | 0 | 0 | 0 | 0 | 34,968* | 39,134* |
| 2XJ | 2015-09-21 | M | 2011 | 0 | 0 | 0 | 0 | 0 | 65,400 | 69,313 |
| 2XL | 2015-09-21 | F | 2011 | 0 | 0 | 0 | 0 | 0 | 88,409* | 105,612* |
| 3AU | 2015-09-21 | F | 2009 | 0 | 0 | 0 | 0 | 0 | 88,885 | 105,388 |
| 40J | 2015-09-21 | F | 2003 | 0 | 0 | 0 | 0 | 0 | 34,133* | 66,420* |
| 257 | 2015-09-22 | F | 2007 | 0 | 0 | 0 | 0 | 0 | 56,005* | 66,278* |
| 3AX | 2016-06-14 | M | 2012 | 0 | 0 | 0 | 0 | 0 | 0 | 183,581 |
| 997 | 2016-06-14 | F | 2003 | 0 | 0 | 0 | 0 | 0 | 0 | 145,984* |
