# Supplementary material for: Vultures and Livestock: The Where, When, and Why of Visits to Farms
Source: Animals (Basel). 2020 Nov 16;10(11):2127. doi: 10.3390/ani10112127 (PMC7698296; doi:10.3390/ani10112127)

Figure S1. Percentage of the study period per each GPS-tagged Egyptian Vulture with locations collected at different time intervals (i.e. time between two consecutive locations). Periods of time corresponding to the night are not shown.

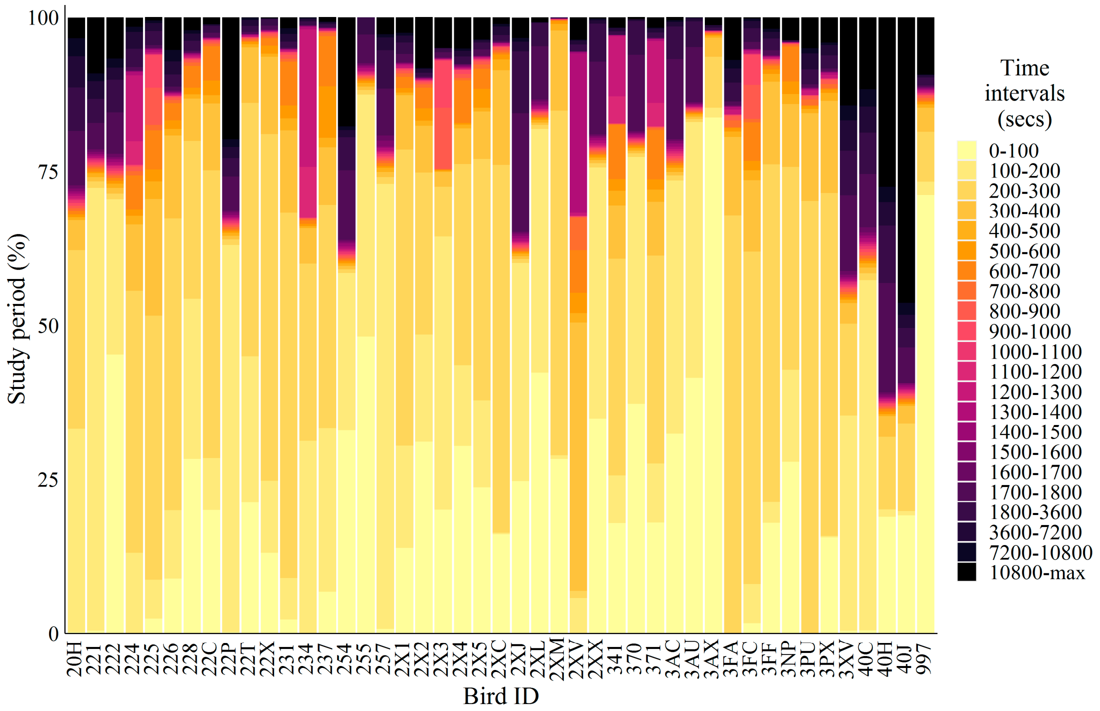

Supplement: Supplementary file 1 [file animals-10-02127-s001.zip › supplementary 2_Figure S1.pdf]
