# Supplementary material for: Vultures and Livestock: The Where, When, and Why of Visits to Farms
Source: Animals (Basel). 2020 Nov 16;10(11):2127. doi: 10.3390/ani10112127 (PMC7698296; doi:10.3390/ani10112127)
Supplement: Supplementary file 1 [file animals-10-02127-s001.zip › supplementary 3_Table S2-4.pdf]

For assessing the influence of random factors, and following suggestions of [1], we compared models including only the intercept, models with one, two or three random factors (noted as 1|Farm ID, 1|Semester ID and 1|Bird ID), and additionally such combinations of random factors plus all selected top ranked combination of variables (Table S8, S9 and S10) . Based on AICc, the best models included two random factors for *FARMS* (Table S2) and three for *VULTURES* (Tables S3 and S4).

Table S2. Modeling the importance of random factors for models with the response variable *FARMS*.

Model	Variables	K	AICc	ΔAICc
mod1	Breeding + Carcass + Goat Sheep + Dist HPFP + Dist Road + Breeding:Dist HPFP + (1|Farm ID) + (1|Semester ID)	9	4415.43	0.00
mod2	(1|Farm ID) + (1|Semester ID)	3	4534.90	119.47
mod3	Breeding + Carcass + Goat Sheep + Dist HPFP + Dist Road + Breeding:Dist HPFP + (1|Farm ID)	8	4555.00	139.56
mod4	(1|Farm ID)	2	4719.58	304.15
mod5	Breeding + Carcass + Goat Sheep + Dist HPFP + Dist Road + Breeding:Dist HPFP + (1|Semester ID)	8	8453.50	4038.07
mod6	Breeding + Carcass + Goat Sheep + Dist HPFP + Dist Road + Breeding:Dist HPFP	7	8590.49	4175.06
mod7	(1|Semester ID)	2	10244.65	5829.22
mod8	1	1	10396.74	5981.30

Table S3. Modeling the importance of random factors for models with the response variable *non-territorial VULTURES*.

Model	Variables	K	AICc	ΔAICc
mod1	Age + AreaK95 + Breeding + Carcass + Goat Sheep + Dist K50 + Dist Road + Dist Terr + Breeding:Dist Terr + (1ǀBird ID) + (1ǀFarm ID) + (1ǀSemester ID)	13	19838.19	0.00
mod2	AreaK95 + Breeding + Carcass + Goat Sheep + Dist K50 + Dist Road + Dist Terr + Breeding:Dist Terr + (1ǀBird ID) + (1ǀFarm ID) + (1ǀSemester ID)	12	19838.28	0.09
mod3	(1ǀBird ID) + (1ǀFarm ID) + (1ǀSemester ID) + Age + AreaK95 + Breeding + Goat Sheep + Dist K50 + Dist Road + Dist Terr + Breeding:Dist Terr	12	19839.15	0.96
mod4	(1ǀBird ID) + (1ǀFarm ID) + (1ǀSemester ID) + AreaK95 + Breeding + Goat Sheep + Dist K50 + Dist Road + Dist Terr + Breeding:Dist Terr	11	19839.25	1.06
mod5	Age + AreaK95 + Breeding + Carcass + Goat Sheep + Dist K50 + Dist Road + Dist Terr + Breeding:Dist Terr + (1ǀBird ID) + (1ǀFarm ID)	12	19863.85	25.66
mod6	Age + AreaK95 + Breeding + Goat Sheep + Dist K50 + Dist Road + Dist Terr + Breeding:Dist Terr + (1ǀBird ID) + (1ǀFarm ID)	11	19864.74	26.55
mod7	AreaK95 + Breeding + Carcass + Goat Sheep + Dist K50 + Dist Road + Dist Terr + Breeding:Dist Terr + (1ǀBird ID) + (1ǀFarm ID)	11	19864.90	26.71
mod8	AreaK95 + Breeding + Goat Sheep + Dist K50 + Dist Road + Dist Terr + Breeding:Dist Terr + (1ǀBird ID) + (1ǀFarm ID)	10	19865.82	27.63
mod9	AreaK95 + Breeding + Carcass + Goat Sheep + Dist K50 + Dist Road + Dist Terr + Breeding:Dist Terr + (1ǀFarm ID) + (1ǀSemester ID)	11	21220.19	1381.99

Model	Variables	K	AICc	ΔAICc
mod10	AreaK95 + Breeding + Goat Sheep + Dist K50 + Dist Road + Dist Terr + Breeding:Dist Terr + (1∣Farm ID) + (1∣Semester ID)	10	21220.96	1382.77
mod11	Age + AreaK95 + Breeding + Carcass + Goat Sheep + Dist K50 + Dist Road + Dist Terr + Breeding:Dist Terr + (1∣Farm ID) + (1∣Semester ID)	12	21222.19	1384.00
mod12	Age + AreaK95 + Breeding + Goat Sheep + Dist K50 + Dist Road + Dist Terr + Breeding:Dist Terr + (1∣Farm ID) + (1∣Semester ID)	11	21222.96	1384.77
mod13	AreaK95 + Breeding + Carcass + Goat Sheep + Dist K50 + Dist Road + Dist Terr + Breeding:Dist Terr + (1∣Farm ID)	10	21319.62	1481.42
mod14	Age + AreaK95 + Breeding + Carcass + Goat Sheep + Dist K50 + Dist Road + Dist Terr + Breeding:Dist Terr + (1∣Farm ID)	11	21320.27	1482.08
mod15	AreaK95 + Breeding + Goat Sheep + Dist K50 + Dist Road + Dist Terr + Breeding:Dist Terr + (1∣Farm ID)	9	21320.30	1482.11
mod16	Age + AreaK95 + Breeding + Goat Sheep + Dist K50 + Dist Road + Dist Terr + Breeding:Dist Terr + (1∣Farm ID)	10	21320.91	1482.72
mod17	(1∣Bird ID) + (1∣Farm ID) + (1∣Semester ID)	4	23521.86	3683.67
mod18	(1∣Farm ID) + (1∣Bird ID)	3	23812.74	3974.55
mod19	(1∣Farm ID) + (1∣Semester ID)	3	25836.65	5998.46
mod20	(1∣Farm ID)	2	26186.79	6348.60
mod21	AreaK95 + Breeding + Carcass + Goat Sheep + Dist K50 + Dist Road + Dist Terr + Breeding:Dist Terr + (1∣Bird ID) + (1∣Semester ID)	11	41028.79	21190.60

Model	Variables	K	AICc	ΔAICc
mod22	Age + AreaK95 + Breeding + Carcass + Goat Sheep + Dist K50 + Dist Road + Dist Terr + Breeding:Dist Terr + (1|Bird ID) + (1|Semester ID)	12	41029.74	21191.54
mod23	AreaK95 + Breeding + Goat Sheep + Dist K50 + Dist Road + Dist Terr + Breeding:Dist Terr + (1|Bird ID) + (1|Semester ID)	10	42107.96	22269.77
mod24	Age + AreaK95 + Breeding + Goat Sheep + Dist K50 + Dist Road + Dist Terr + Breeding:Dist Terr + (1|Bird ID) + (1|Semester ID)	11	42109.91	22271.72
mod25	AreaK95 + Breeding + Carcass + Goat Sheep + Dist K50 + Dist Road + Dist Terr + Breeding:Dist Terr + (1|Semester ID)	10	42478.21	22640.02
mod26	Age + AreaK95 + Breeding + Carcass + Goat Sheep + Dist K50 + Dist Road + Dist Terr + Breeding:Dist Terr + (1|Semester ID)	11	42479.03	22640.83
mod27	Age + AreaK95 + Breeding + Goat Sheep + Dist K50 + Dist Road + Dist Terr + Breeding:Dist Terr + (1|Semester ID)	10	43623.01	23784.82
mod28	AreaK95 + Breeding + Goat Sheep + Dist K50 + Dist Road + Dist Terr + Breeding:Dist Terr + (1|Semester ID)	9	43624.89	23786.70
mod29	(1|Semester ID) + (1|Bird ID)	3	47558.38	27720.19
mod30	(1|Bird ID)	2	48036.32	28198.13
mod31	(1|Semester ID)	2	49883.80	30045.61
mod32	1	1	50549.02	30710.83

Table S4. Modeling the importance of random factors for models with the response variable *territorial VULTURES*.

Model	Variables	K	AICc	ΔAICc
mod1	Age + AreaK95 + Goat Sheep + Dist K50 + Dist HPFP + Dist Road + Dist Terr + Sex + Age:Dist K50 + (1∣Bird ID) + (1∣Farm ID) + (1∣Semester ID)	13	6786.54	0.00
mod2	Age + AreaK95 + Goat Sheep + Dist K50 + Dist Road + Dist Terr + Sex + Age:Dist K50 + (1∣Bird ID) + (1∣Farm ID) + (1∣Semester ID)	12	6789.44	2.90
mod3	Age + AreaK95 + Breeding + Goat Sheep + Dist K50 + Dist HPFP + Dist Road + Dist Terr + Age:Dist K50 + (1∣Bird ID) + (1∣Farm ID) + (1∣Semester ID)	13	6789.60	3.06
mod4	Age + AreaK95 + Goat Sheep + Dist K50 + Dist HPFP + Dist Road + Dist Terr + Age:Dist K50 + (1∣Bird ID) + (1∣Farm ID) + (1∣Semester ID)	12	6789.60	3.06
mod5	Age + AreaK95 + Goat Sheep + Dist K50 + Dist HPFP + Dist Terr + Sex + Age:Dist K50 + (1∣Bird ID) + (1∣Farm ID) + (1∣Semester ID)	12	6790.64	4.10
mod6	Age + AreaK95 + Goat Sheep + Dist K50 + Dist Road + Dist Terr + Age:Dist K50 + (1∣Bird ID) + (1∣Farm ID) + (1∣Semester ID)	11	6792.52	5.98
mod7	Age + AreaK95 + Breeding + Goat Sheep + Dist K50 + Dist Road + Dist Terr + Age:Dist K50 + (1∣Bird ID) + (1∣Farm ID) + (1∣Semester ID)	12	6792.52	5.98
mod8	Age + AreaK95 + Carcass + Goat Sheep + Dist K50 + Dist Terr + Sex + Age:Dist K50 + (1∣Bird ID) + (1∣Farm ID) + (1∣Semester ID)	12	6793.52	6.98
mod9	Age + AreaK95 + Goat Sheep + Dist K50 + Dist HPFP + Dist Terr + Age:Dist K50 + (1∣Bird ID) + (1∣Farm ID) + (1∣Semester ID)	11	6793.74	7.20

Model	Variables	K	AICc	ΔAICc
mod10	Age + AreaK95 + Breeding + Goat Sheep + Dist K50 + Dist HPFP + Dist Terr + Age:Dist K50 + (1∣Bird ID) + (1∣Farm ID) + (1∣Semester ID)	12	6793.75	7.20
mod11	Age + AreaK95 + Goat Sheep + Dist K50 + Dist Terr + Sex + Age:Dist K50 + (1∣Bird ID) + (1∣Farm ID) + (1∣Semester ID)	11	6794.48	7.93
mod12	Age + AreaK95 + Carcass + Goat Sheep + Dist K50 + Dist Terr + Age:Dist K50 + (1∣Bird ID) + (1∣Farm ID) + (1∣Semester ID)	11	6796.64	10.10
mod13	Age + AreaK95 + Breeding + Carcass + Goat Sheep + Dist K50 + Dist Terr + Age:Dist K50 + (1∣Bird ID) + (1∣Farm ID) + (1∣Semester ID)	12	6796.66	10.11
mod14	Age + AreaK95 + Goat Sheep + Dist K50 + Dist Terr + Age:Dist K50 + (1∣Bird ID) + (1∣Farm ID) + (1∣Semester ID)	10	6797.60	11.06
mod15	Age + AreaK95 + Breeding + Goat Sheep + Dist K50 + Dist Terr + Age:Dist K50 + (1∣Bird ID) + (1∣Farm ID) + (1∣Semester ID)	11	6797.61	11.07
mod16	Age + AreaK95 + Breeding + Goat Sheep + Dist K50 + Dist HPFP + Dist Road + Dist Terr + Age:Dist K50 + (1∣Bird ID) + (1∣Farm ID)	12	6833.69	47.15
mod17	Age + AreaK95 + Breeding + Goat Sheep + Dist K50 + Dist Road + Dist Terr + Age:Dist K50 + (1∣Bird ID) + (1∣Farm ID)	11	6837.36	50.81
mod18	Age + AreaK95 + Breeding + Goat Sheep + Dist K50 + Dist HPFP + Dist Terr + Age:Dist K50 + (1∣Bird ID) + (1∣Farm ID)	11	6838.07	51.53
mod19	Age + AreaK95 + Breeding + Carcass + Goat Sheep + Dist K50 + Dist Terr + Age:Dist K50 + (1∣Bird ID) + (1∣Farm ID)	11	6841.60	55.06

Model	Variables	K	AICc	ΔAICc
mod20	Age + AreaK95 + Breeding + Goat Sheep + Dist K50 + Dist Terr + Age:Dist K50 + (1∣Bird ID) + (1∣Farm ID)	10	6842.74	56.20
mod21	Age + AreaK95 + Goat Sheep + Dist K50 + Dist HPFP + Dist Road + Dist Terr + Sex + Age:Dist K50 + (1∣Bird ID) + (1∣Farm ID)	12	6989.27	202.72
mod22	Age + AreaK95 + Goat Sheep + Dist K50 + Dist HPFP + Dist Road + Dist Terr + Age:Dist K50 + (1∣Bird ID) + (1∣Farm ID)	11	6989.45	202.91
mod23	Age + AreaK95 + Goat Sheep + Dist K50 + Dist HPFP + Dist Terr + Sex + Age:Dist K50 + (1∣Bird ID) + (1∣Farm ID)	11	6993.89	207.35
mod24	Age + AreaK95 + Goat Sheep + Dist K50 + Dist HPFP + Dist Terr + Age:Dist K50 + (1∣Bird ID) + (1∣Farm ID)	10	6994.08	207.53
mod25	Age + AreaK95 + Goat Sheep + Dist K50 + Dist Road + Dist Terr + Sex + Age:Dist K50 + (1∣Bird ID) + (1∣Farm ID)	11	6994.08	207.53
mod26	Age + AreaK95 + Goat Sheep + Dist K50 + Dist Road + Dist Terr + Age:Dist K50 + (1∣Bird ID) + (1∣Farm ID)	10	6994.26	207.71
mod27	Age + AreaK95 + Carcass + Goat Sheep + Dist K50 + Dist Terr + Sex + Age:Dist K50 + (1∣Bird ID) + (1∣Farm ID)	11	6998.24	211.70
mod28	Age + AreaK95 + Carcass + Goat Sheep + Dist K50 + Dist Terr + Age:Dist K50 + (1∣Bird ID) + (1∣Farm ID)	10	6998.43	211.88
mod29	Age + AreaK95 + Goat Sheep + Dist K50 + Dist Terr + Sex + Age:Dist K50 + (1∣Bird ID) + (1∣Farm ID)	10	6999.81	213.27

Model	Variables	K	AICc	ΔAICc
mod30	Age + AreaK95 + Goat Sheep + Dist K50 + Dist Terr + Age:Dist K50 + (1|Bird ID) + (1|Farm ID)	9	6999.99	213.45
mod31	Age + AreaK95 + Goat Sheep + Dist K50 + Dist HPFP + Dist Road + Dist Terr + Sex + Age:Dist K50 + (1|Farm ID) + (1|Semester ID)	12	7277.93	491.39
mod32	Age + AreaK95 + Goat Sheep + Dist K50 + Dist Road + Dist Terr + Sex + Age:Dist K50 + (1|Farm ID) + (1|Semester ID)	11	7278.82	492.27
mod33	Age + AreaK95 + Goat Sheep + Dist K50 + Dist HPFP + Dist Terr + Sex + Age:Dist K50 + (1|Farm ID) + (1|Semester ID)	11	7279.54	493.00
mod34	Age + AreaK95 + Carcass + Goat Sheep + Dist K50 + Dist Terr + Sex + Age:Dist K50 + (1|Farm ID) + (1|Semester ID)	11	7280.51	493.97
mod35	Age + AreaK95 + Goat Sheep + Dist K50 + Dist Terr + Sex + Age:Dist K50 + (1|Farm ID) + (1|Semester ID)	10	7281.07	494.52
mod36	Age + AreaK95 + Breeding + Goat Sheep + Dist K50 + Dist HPFP + Dist Road + Dist Terr + Age:Dist K50 + (1|Farm ID) + (1|Semester ID)	12	7524.17	737.63
mod37	Age + AreaK95 + Breeding + Goat Sheep + Dist K50 + Dist Road + Dist Terr + Age:Dist K50 + (1|Farm ID) + (1|Semester ID)	11	7524.19	737.64
mod38	Age + AreaK95 + Goat Sheep + Dist K50 + Dist HPFP + Dist Road + Dist Terr + Age:Dist K50 + (1|Farm ID) + (1|Semester ID)	11	7525.73	739.19
mod39	Age + AreaK95 + Goat Sheep + Dist K50 + Dist Road + Dist Terr + Age:Dist K50 + (1|Farm ID) + (1|Semester ID)	10	7525.76	739.21

Model	Variables	K	AICc	ΔAICc
mod40	Age + AreaK95 + Breeding + Goat Sheep + Dist K50 + Dist HPFP + Dist Terr + Age:Dist K50 + (1∣Farm ID) + (1∣Semester ID)	11	7525.84	739.29
mod41	Age + AreaK95 + Breeding + Carcass + Goat Sheep + Dist K50 + Dist Terr + Age:Dist K50 + (1∣Farm ID) + (1∣Semester ID)	11	7526.06	739.51
mod42	Age + AreaK95 + Breeding + Goat Sheep + Dist K50 + Dist Terr + Age:Dist K50 + (1∣Farm ID) + (1∣Semester ID)	10	7526.42	739.88
mod43	Age + AreaK95 + Goat Sheep + Dist K50 + Dist HPFP + Dist Terr + Age:Dist K50 + (1∣Farm ID) + (1∣Semester ID)	10	7527.38	740.83
mod44	Age + AreaK95 + Carcass + Goat Sheep + Dist K50 + Dist Terr + Age:Dist K50 + (1∣Farm ID) + (1∣Semester ID)	10	7527.58	741.04
mod45	Age + AreaK95 + Goat Sheep + Dist K50 + Dist Terr + Age:Dist K50 + (1∣Farm ID) + (1∣Semester ID)	9	7527.97	741.42
mod46	Age + AreaK95 + Goat Sheep + Dist K50 + Dist HPFP + Dist Road + Dist Terr + Sex + Age:Dist K50 + (1∣Farm ID)	11	7556.45	769.91
mod47	Age + AreaK95 + Goat Sheep + Dist K50 + Dist Road + Dist Terr + Sex + Age:Dist K50 + (1∣Farm ID)	10	7557.78	771.23
mod48	Age + AreaK95 + Goat Sheep + Dist K50 + Dist HPFP + Dist Terr + Sex + Age:Dist K50 + (1∣Farm ID)	10	7560.19	773.64
mod49	Age + AreaK95 + Carcass + Goat Sheep + Dist K50 + Dist Terr + Sex + Age:Dist K50 + (1∣Farm ID)	10	7561.59	775.04

Model	Variables	K	AICc	ΔAICc
mod50	Age + AreaK95 + Goat Sheep + Dist K50 + Dist Terr + Sex + Age:Dist K50 + (1|Farm ID)	9	7562.37	775.83
mod51	Age + AreaK95 + Breeding + Goat Sheep + Dist K50 + Dist HPFP + Dist Road + Dist Terr + Age:Dist K50 + (1|Farm ID)	11	7570.78	784.24
mod52	Age + AreaK95 + Breeding + Goat Sheep + Dist K50 + Dist Road + Dist Terr + Age:Dist K50 + (1|Farm ID)	10	7570.86	784.32
mod53	Age + AreaK95 + Breeding + Goat Sheep + Dist K50 + Dist HPFP + Dist Terr + Age:Dist K50 + (1|Farm ID)	10	7572.96	786.42
mod54	Age + AreaK95 + Breeding + Carcass + Goat Sheep + Dist K50 + Dist Terr + Age:Dist K50 + (1|Farm ID)	10	7573.27	786.73
mod55	Age + AreaK95 + Breeding + Goat Sheep + Dist K50 + Dist Terr + Age:Dist K50 + (1|Farm ID)	9	7573.65	787.11
mod56	Age + AreaK95 + Goat Sheep + Dist K50 + Dist HPFP + Dist Road + Dist Terr + Age:Dist K50 + (1|Farm ID)	10	7754.20	967.66
mod57	Age + AreaK95 + Goat Sheep + Dist K50 + Dist Road + Dist Terr + Age:Dist K50 + (1|Farm ID)	9	7754.72	968.18
mod58	Age + AreaK95 + Goat Sheep + Dist K50 + Dist HPFP + Dist Terr + Age:Dist K50 + (1|Farm ID)	9	7757.09	970.54
mod59	Age + AreaK95 + Carcass + Goat Sheep + Dist K50 + Dist Terr + Age:Dist K50 + (1|Farm ID)	9	7757.73	971.18

Model	Variables	K	AICc	ΔAICc
mod60	Age + AreaK95 + Goat Sheep + Dist K50 + Dist Terr + Age:Dist K50 + (1 ∣ Farm ID)	8	7758.34	971.80
mod61	(1 ∣ Bird ID) + (1 ∣ Farm ID) + (1 ∣ Semester ID)	4	8926.83	2140.28
mod62	(1 ∣ Farm ID) + (1 ∣ Bird ID)	3	9261.70	2475.16
mod63	(1 ∣ Farm ID) + (1 ∣ Semester ID)	3	14336.30	7549.75
mod64	(1 ∣ Farm ID)	2	14706.01	7919.47
mod65	Age + AreaK95 + Goat Sheep + Dist K50 + Dist HPFP + Dist Road + Dist Terr + Age:Dist K50 + (1 ∣ Bird ID) + (1 ∣ Semester ID)	11	18695.79	11909.25
mod66	Age + AreaK95 + Goat Sheep + Dist K50 + Dist HPFP + Dist Road + Dist Terr + Sex + Age:Dist K50 + (1 ∣ Bird ID) + (1 ∣ Semester ID)	12	18696.99	11910.45
mod67	Age + AreaK95 + Breeding + Goat Sheep + Dist K50 + Dist HPFP + Dist Road + Dist Terr + Age:Dist K50 + (1 ∣ Bird ID) + (1 ∣ Semester ID)	12	18697.55	11911.00
mod68	Age + AreaK95 + Goat Sheep + Dist K50 + Dist HPFP + Dist Terr + Age:Dist K50 + (1 ∣ Bird ID) + (1 ∣ Semester ID)	10	18770.03	11983.49
mod69	Age + AreaK95 + Goat Sheep + Dist K50 + Dist HPFP + Dist Terr + Sex + Age:Dist K50 + (1 ∣ Bird ID) + (1 ∣ Semester ID)	11	18771.38	11984.83
mod70	Age + AreaK95 + Breeding + Goat Sheep + Dist K50 + Dist HPFP + Dist Terr + Age:Dist K50 + (1 ∣ Bird ID) + (1 ∣ Semester ID)	11	18771.89	11985.34

Model	Variables	K	AICc	ΔAICc
mod71	Age + AreaK95 + Goat Sheep + Dist K50 + Dist Road + Dist Terr + Age:Dist K50 + (1|Bird ID) + (1|Semester ID)	10	18983.74	12197.19
mod72	Age + AreaK95 + Breeding + Goat Sheep + Dist K50 + Dist Road + Dist Terr + Age:Dist K50 + (1|Bird ID) + (1|Semester ID)	11	18984.98	12198.44
mod73	Age + AreaK95 + Goat Sheep + Dist K50 + Dist Road + Dist Terr + Sex + Age:Dist K50 + (1|Bird ID) + (1|Semester ID)	11	18985.23	12198.69
mod74	Age + AreaK95 + Carcass + Goat Sheep + Dist K50 + Dist Terr + Age:Dist K50 + (1|Bird ID) + (1|Semester ID)	10	19041.03	12254.49
mod75	Age + AreaK95 + Breeding + Carcass + Goat Sheep + Dist K50 + Dist Terr + Age:Dist K50 + (1|Bird ID) + (1|Semester ID)	11	19042.45	12255.91
mod76	Age + AreaK95 + Carcass + Goat Sheep + Dist K50 + Dist Terr + Sex + Age:Dist K50 + (1|Bird ID) + (1|Semester ID)	11	19042.65	12256.11
mod77	Age + AreaK95 + Goat Sheep + Dist K50 + Dist Terr + Age:Dist K50 + (1|Bird ID) + (1|Semester ID)	9	19048.82	12262.28
mod78	Age + AreaK95 + Breeding + Goat Sheep + Dist K50 + Dist Terr + Age:Dist K50 + (1|Bird ID) + (1|Semester ID)	10	19050.19	12263.64
mod79	Age + AreaK95 + Goat Sheep + Dist K50 + Dist Terr + Sex + Age:Dist K50 + (1|Bird ID) + (1|Semester ID)	10	19050.47	12263.93
mod80	Age + AreaK95 + Goat Sheep + Dist K50 + Dist HPFP + Dist Road + Dist Terr + Sex + Age:Dist K50 + (1|Semester ID)	11	21356.72	14570.17

Model	Variables	K	AICc	ΔAICc
mod81	Age + AreaK95 + Goat Sheep + Dist K50 + Dist HPFP + Dist Terr + Sex + Age:Dist K50 + (1 | Semester ID)	10	21396.02	14609.47
mod82	Age + AreaK95 + Goat Sheep + Dist K50 + Dist HPFP + Dist Road + Dist Terr + Age:Dist K50 + (1 | Semester ID)	10	21446.46	14659.91
mod83	Age + AreaK95 + Breeding + Goat Sheep + Dist K50 + Dist HPFP + Dist Road + Dist Terr + Age:Dist K50 + (1 | Semester ID)	11	21447.69	14661.15
mod84	Age + AreaK95 + Goat Sheep + Dist K50 + Dist Road + Dist Terr + Sex + Age:Dist K50 + (1 | Semester ID)	10	21462.65	14676.11
mod85	Age + AreaK95 + Goat Sheep + Dist K50 + Dist HPFP + Dist Terr + Age:Dist K50 + (1 | Semester ID)	9	21466.22	14679.68
mod86	Age + AreaK95 + Breeding + Goat Sheep + Dist K50 + Dist HPFP + Dist Terr + Age:Dist K50 + (1 | Semester ID)	10	21467.49	14680.94
mod87	Age + AreaK95 + Carcass + Goat Sheep + Dist K50 + Dist Terr + Sex + Age:Dist K50 + (1 | Semester ID)	10	21484.94	14698.40
mod88	Age + AreaK95 + Goat Sheep + Dist K50 + Dist Terr + Sex + Age:Dist K50 + (1 | Semester ID)	9	21502.68	14716.14
mod89	Age + AreaK95 + Goat Sheep + Dist K50 + Dist Road + Dist Terr + Age:Dist K50 + (1 | Semester ID)	9	21515.96	14729.42
mod90	Age + AreaK95 + Breeding + Goat Sheep + Dist K50 + Dist Road + Dist Terr + Age:Dist K50 + (1 | Semester ID)	10	21516.95	14730.41

Model	Variables	K	AICc	ΔAICc
mod91	Age + AreaK95 + Carcass + Goat Sheep + Dist K50 + Dist Terr + Age:Dist K50 + (1|Semester ID)	9	21527.91	14741.36
mod92	Age + AreaK95 + Breeding + Carcass + Goat Sheep + Dist K50 + Dist Terr + Age:Dist K50 + (1|Semester ID)	10	21529.01	14742.47
mod93	Age + AreaK95 + Goat Sheep + Dist K50 + Dist Terr + Age:Dist K50 + (1|Semester ID)	8	21539.46	14752.92
mod94	Age + AreaK95 + Breeding + Goat Sheep + Dist K50 + Dist Terr + Age:Dist K50 + (1|Semester ID)	9	21540.47	14753.93
mod95	(1|Semester ID) + (1|Bird ID)	3	26445.46	19658.92
mod96	(1|Bird ID)	2	26948.82	20162.28
mod97	(1|Semester ID)	2	33781.05	26994.50
mod98	1	1	33903.49	27116.95

Referencces

1. Zuur, A.F.; Ieno, E.N.; Walker, N.J.; Savelieve, A.A.; Smith, G.M. *Mixed effects models and extensions in ecology with R*; Gails, M., Krickeberg, K., Samet, J.M., Tsiatis, A., Wong, W., Eds.; Springer, 2009; ISBN 978-0-387-87457-9.
