# Supplementary material for: Vultures and Livestock: The Where, When, and Why of Visits to Farms
Source: Animals (Basel). 2020 Nov 16;10(11):2127. doi: 10.3390/ani10112127 (PMC7698296; doi:10.3390/ani10112127)
Supplement: Supplementary file 1 [file animals-10-02127-s001.zip › supplementary 4_Table S5.pdf]

Table S5. Variance inflation factors (VIF) for explanatory variables in the datasets used for modeling the drivers of Egyptian Vulture visits to livestock farms in Fuerteventura (Canary Island). Each "case" represents a group of variables in which no pair violates the assumption of independence based on Spearman's correlation coefficient lower than |0.5|. Only variables included in a same case were included in the same model, avoiding collinearity problems [1]. VIF was calculated for the combination of variables in each case.

Variables	Farms		Non-territorial		Territorial			
	Case 1	Case 2	Case 1	Case 2	Case 1	Case 2	Case 3	Case 4
Age	-	-	1.05	1.05	1.06	1.10	1.06	1.10
AreaK95	-	-	1.13	1.12	-	1.10	-	1.09
Carcass	-	1.14	-	1.11	-	-	1.15	1.15
Dist K50	-	-	1.11	1.13	1.22	1.09	1.23	1.10
Dist Nest	-	-	-	-	1.24	-	1.24	-
Dist HPFP	1.01	1.07	1.12	1.16	1.11	1.08	1.15	1.12
Dist Road	-	1.12	-	1.10	1.28	1.28	1.09	1.09
Dist Terr	-	-	1.10	1.10	1.11	1.10	1.10	1.10
Dist Urb	1.01	-	1.06	-	1.31	1.31	-	-
Goat Sheep	1.01	1.01	1.01	1.01	1.02	1.02	1.01	1.01

References

1. Graham, M.H. Statistical confronting multicollinearity in ecological. *Ecology* **2003**, *84*, 2809–2815.
