# Supplementary material for: Vultures and Livestock: The Where, When, and Why of Visits to Farms
Source: Animals (Basel). 2020 Nov 16;10(11):2127. doi: 10.3390/ani10112127 (PMC7698296; doi:10.3390/ani10112127)
Supplement: Supplementary file 1 [file animals-10-02127-s001.zip › supplementary 5_Table S6-7.pdf]

Table S6. Models without uninformative parameters predicting the probability of carcass abandonment in each farm in Fuerteventura (Canary Island). ΔAICc: difference in AICc between each model and the best model (lowest AICc). wi: Akaike weights. Cum. wi: Cumulative Akaike weights. K: number of parameters. Exp. Deviance: adjusted explained deviance [1].

Model	Variable	AICc	ΔAICc	wi	Cum. wi	K	Exp. Deviance
mod1	Dist Dump + Dist Urb	89.71	0.000	0.794	0.79	3	16.8
mod2	Dist Urb	92.83	3.118	0.167	0.96	2	12.7
mod3	Dist Dump + Dist road	96.64	6.926	0.025	0.99	3	9.9
mod4	Dist Dump	98.45	8.740	0.010	1.00	2	7.2
mod5	Dist road	100.37	10.653	0.004	1.00	2	5.3
mod0	1	105.01	15.301	0.000	1.00	1	0

Table S7. Estimates and standard errors from the best model (lowest AICc) for variable *Carcass*. 85% confidence intervals of the estimates are also shown (7.5% and 92.5% limits). Note that models were fitted with the explanatory variables at the original scale and estimates are the original outcome.

Variable	Estimate	Std. Error	7.5 %	92.5 %
(Intercept)	-1.54568	0.57865	-2.42778	-0.75038
Dist Dump	0.00005	0.00002	0.00002	0.00008
Dist Urb	0.00087	0.00030	0.00047	0.00132

References

1. Guisan, A.; Zimmermann, N.E. Predictive habitat distribution models in ecology. *Ecol. Modell.* **2000**, *135*, 147–186, doi:10.1016/S0304-3800(00)00354-9.
