# Supplementary material for: Vultures and Livestock: The Where, When, and Why of Visits to Farms
Source: Animals (Basel). 2020 Nov 16;10(11):2127. doi: 10.3390/ani10112127 (PMC7698296; doi:10.3390/ani10112127)

Figure S2. Box-plots showing the differential use of farms by Egyptian Vultures according to sex, territorial status and breeding season. The line within boxes indicates the median, the edges of the boxes the first (Q1) and third (Q3) quartiles, and the whiskers extend 1.5 times the interquartile range. N.farms HR: number of farms included in individual home range; N.farms visited: number of farms visited; Goat Sheep visited: mean number of goats and sheep in visited farms; N.days farms: number of days with visits to farms; Dist HPFP: distance (km) to highly predictable feeding places for visited farms; AreaK95: area of individual home ranges (KernelUD 95%, km^2); AreaK50: area of individual core areas (KernelUD 50%, km^2); Number of data: number of semesters of individuals.

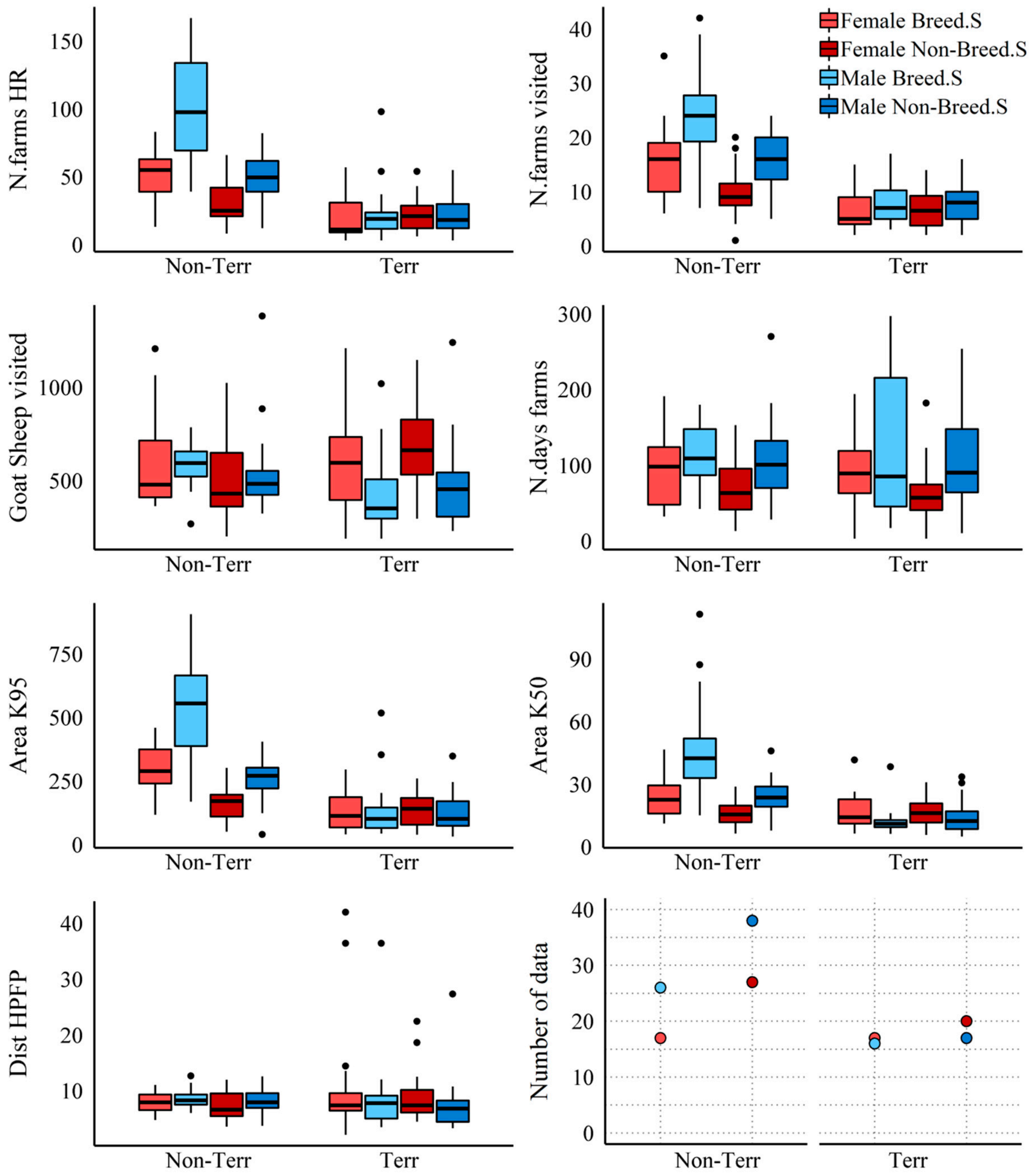

Supplement: Supplementary file 1 [file animals-10-02127-s001.zip › supplementary 6_Figure S2.pdf]
