# Supplementary material for: Vultures and Livestock: The Where, When, and Why of Visits to Farms
Source: Animals (Basel). 2020 Nov 16;10(11):2127. doi: 10.3390/ani10112127 (PMC7698296; doi:10.3390/ani10112127)

The mean daily distance ± SD between all Egyptian Vultures' locations selected as visits to farms and the nearest farm was 88.0 ± 41.1 m (N = 18999). Values were very similar when separating non-territorial and territorial females (84.7 ± 39 m, N = 3826 and 84.1 ± 43.1 m, N = 3205) and males (85.5 ± 40.1 m, N = 7562 and 98.0 ± 41.2 m, N = 4406) respectively.

Figure S3. Frequency distribution of distance (m) from GPS-locations to farms considering a) all locations used to analyze the use of farms by vultures; the daily mean calculated by vulture and farm for b) all the individuals, c) territorial females, d) non-territorial females, e) territorial males and f) non-territorial males.

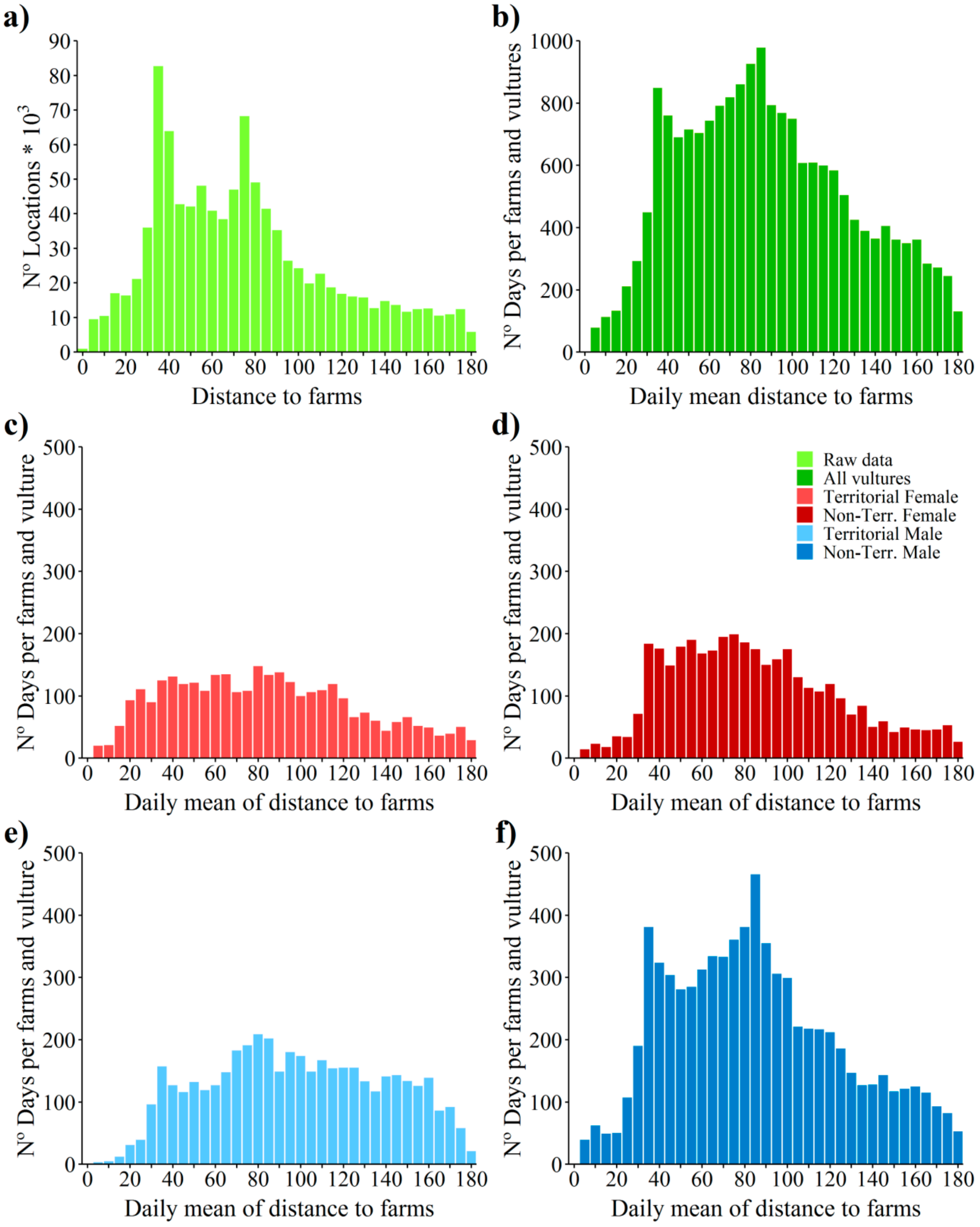

Supplement: Supplementary file 1 [file animals-10-02127-s001.zip › supplementary 7_Figure S3.pdf]
