# Supplementary material for: Vultures and Livestock: The Where, When, and Why of Visits to Farms
Source: Animals (Basel). 2020 Nov 16;10(11):2127. doi: 10.3390/ani10112127 (PMC7698296; doi:10.3390/ani10112127)
Supplement: Supplementary file 1 [file animals-10-02127-s001.zip › supplementary 8_Table S8-10.pdf]

Table S8. Top-ranked models assessing drivers of the selection of farms by Canarian Egyptian Vultures for the response variable *FARMS*. See Table 2 for a full description of explanatory variables. The number of parameters (K), the AICc, the difference in AICc between each model and the best model (ΔAICc), and Akaike weights (wi) are shown. "1|Farm ID" and "1|Semester ID" indicate the random effect of farm and Semester identity respectively. Only the 20 highest-ranked and the null (intercept plus random terms) models are shown. All combinations from only the intercept to all considered fixed effects were tested.

Model	Variable	K	AICc	ΔAICc	wi
mod1	Breeding + Breeding:Dist HPFP + Carcass + Goat Sheep + Dist HPFP + Dist Road + (1|Farm ID) + (1|Semester ID)	9	4415.43	0.000	0.994
mod2	Breeding + Carcass + Carcass:Dist Road + Goat Sheep + Dist HPFP + Dist Road + (1|Farm ID) + (1|Semester ID)	9	4427.15	11.713	0.003
mod3	Carcass + Carcass:Dist Road + Goat Sheep + Dist HPFP + Dist Road + (1|Farm ID) + (1|Semester ID)	8	4427.83	12.398	0.002
mod4	Breeding + Breeding:Dist HPFP + Goat Sheep + Dist HPFP + Dist Road + (1|Farm ID) + (1|Semester ID)	8	4430.24	14.811	0.001
mod5	Breeding + Carcass + Goat Sheep + Goat Sheep:Dist HPFP + Dist HPFP + Dist Road + (1|Farm ID) + (1|Semester ID)	9	4432.12	16.686	0.000
mod6	Carcass + Goat Sheep + Goat Sheep:Dist HPFP + Dist HPFP + Dist Road + (1|Farm ID) + (1|Semester ID)	8	4432.87	17.434	0.000
mod7	Breeding + Carcass + Goat Sheep + Goat Sheep:Dist Road + Dist HPFP + Dist Road + (1|Farm ID) + (1|Semester ID)	9	4433.46	18.029	0.000
mod8	Carcass + Goat Sheep + Goat Sheep:Dist Road + Dist HPFP + Dist Road + (1|Farm ID) + (1|Semester ID)	8	4434.10	18.665	0.000
mod9	Breeding + Breeding:Dist HPFP + Carcass + Goat Sheep + Dist HPFP + (1|Farm ID) + (1|Semester ID)	8	4434.57	19.136	0.000
mod10	Breeding + Breeding:Goat Sheep + Carcass + Goat Sheep + Dist HPFP + Dist Road + (1|Farm ID) + (1|Semester ID)	9	4435.65	20.217	0.000
mod11	Breeding + Breeding:Carcass + Carcass + Goat Sheep + Dist HPFP + Dist Road + (1|Farm ID) + (1|Semester ID)	9	4436.87	21.435	0.000

Model	Variable	K	AICc	ΔAICc	wi
mod12	Breeding + Breeding:Dist Road + Carcass + Goat Sheep + Dist HPFP + Dist Road + (1|Farm ID) + (1|Semester ID)	9	4437.25	21.820	0.000
mod13	Breeding + Carcass + Goat Sheep + Dist HPFP + Dist Road + (1|Farm ID) + (1|Semester ID)	8	4437.72	22.288	0.000
mod14	Carcass + Goat Sheep + Dist HPFP + Dist Road + (1|Farm ID) + (1|Semester ID)	7	4438.41	22.977	0.000
mod15	Breeding + Breeding:Dist HPFP + Goat Sheep + Dist Urb + Dist HPFP + (1|Farm ID) + (1|Semester ID)	8	4446.67	31.234	0.000
mod16	Breeding + Goat Sheep + Goat Sheep:Dist HPFP + Dist HPFP + Dist Road + (1|Farm ID) + (1|Semester ID)	8	4447.72	32.286	0.000
mod17	Breeding + Goat Sheep + Goat Sheep:Dist Road + Dist HPFP + Dist Road + (1|Farm ID) + (1|Semester ID)	8	4447.85	32.418	0.000
mod18	Breeding + Carcass + Carcass:Dist Road + Goat Sheep + Dist Road + (1|Farm ID) + (1|Semester ID)	8	4447.89	32.454	0.000
mod19	Goat Sheep + Goat Sheep:Dist HPFP + Dist HPFP + Dist Road + (1|Farm ID) + (1|Semester ID)	7	4448.46	33.027	0.000
mod20	Goat Sheep + Goat Sheep:Dist Road + Dist HPFP + Dist Road + (1|Farm ID) + (1|Semester ID)	7	4448.48	33.049	0.000
mod0	(1|Farm ID) + (1|Semester ID)	3	4534.90	119.465	0.000

Table S9. Top-ranked models assessing drivers of the selection of farms by Canarian Egyptian Vultures for the response variable *territorial VULTURES*. See Table 2 for a full description of explanatory variables. The number of parameters (K), the AICc, the difference in AICc between each model and the best model (ΔAICc), and Akaike weights (wi) are shown. "1|Bird ID", "1|Farm ID" and "1|Semester ID" indicate the random effect of bird, farm, and Semester identity respectively. Only the 20 highest-ranked and the null (intercept plus random terms) models are shown. All combinations from only the intercept to all considered fixed effects were tested.

Model	Variable	K	AICc	ΔAICc	wi
mod1	Age + Age:Dist K50 + AreaK95 + Goat Sheep + Dist K50 + Dist HPFP + Dist Road + Dist Terr + Sex + (1|Farm ID) + (1|Bird ID) + (1|Semester ID)	13	6786.54	0.000	0.493
mod2	Age + Age:Dist K50 + AreaK95 + Goat Sheep + Dist K50 + Dist Road + Dist Terr + Sex + (1|Farm ID) + (1|Bird ID) + (1|Semester ID)	12	6789.44	2.896	0.116
mod3	Age + Age:Dist K50 + AreaK95 + Breeding + Goat Sheep + Dist K50 + Dist HPFP + Dist Road + Dist Terr + (1|Farm ID) + (1|Bird ID) + (1|Semester ID)	13	6789.60	3.055	0.107
mod4	Age + Age:Dist K50 + AreaK95 + Goat Sheep + Dist K50 + Dist HPFP + Dist Road + Dist Terr + (1|Farm ID) + (1|Bird ID) + (1|Semester ID)	12	6789.60	3.059	0.107
mod5	Age + Age:Dist K50 + AreaK95 + Goat Sheep + Dist K50 + Dist HPFP + Dist Terr + Sex + (1|Farm ID) + (1|Bird ID) + (1|Semester ID)	12	6790.64	4.099	0.064
mod6	Age + Age:Dist K50 + AreaK95 + Goat Sheep + Dist K50 + Dist Road + Dist Terr + (1|Farm ID) + (1|Bird ID) + (1|Semester ID)	11	6792.52	5.979	0.025
mod7	Age + Age:Dist K50 + AreaK95 + Breeding + Goat Sheep + Dist K50 + Dist Road + Dist Terr + (1|Farm ID) + (1|Bird ID) + (1|Semester ID)	12	6792.52	5.980	0.025
mod8	Age + Age:Dist K50 + AreaK95 + Carcass + Goat Sheep + Dist K50 + Dist Terr + Sex + (1|Farm ID) + (1|Bird ID) + (1|Semester ID)	12	6793.52	6.977	0.015
mod9	Age + Age:Dist K50 + AreaK95 + Goat Sheep + Dist K50 + Dist HPFP + Dist Terr + (1|Farm ID) + (1|Bird ID) + (1|Semester ID)	11	6793.74	7.198	0.013

Model	Variable	K	AICc	ΔAICc	wi
mod10	Age + Age:Dist K50 + AreaK95 + Breeding + Goat Sheep + Dist K50 + Dist HPFP + Dist Terr + (1∣Farm ID) + (1∣Bird ID) + (1∣Semester ID)	12	6793.75	7.204	0.013
mod11	Age + Age:Dist K50 + AreaK95 + Goat Sheep + Dist K50 + Dist Terr + Sex + (1∣Farm ID) + (1∣Bird ID) + (1∣Semester ID)	11	6794.48	7.931	0.009
mod12	Age + Age:Dist K50 + AreaK95 + Carcass + Goat Sheep + Dist K50 + Dist Terr + (1∣Farm ID) + (1∣Bird ID) + (1∣Semester ID)	11	6796.64	10.097	0.003
mod13	Age + Age:Dist K50 + AreaK95 + Breeding + Carcass + Goat Sheep + Dist K50 + Dist Terr + (1∣Farm ID) + (1∣Bird ID) + (1∣Semester ID)	12	6796.66	10.112	0.003
mod14	Age + Age:Dist K50 + AreaK95 + Goat Sheep + Dist K50 + Dist Terr + (1∣Farm ID) + (1∣Bird ID) + (1∣Semester ID)	10	6797.60	11.059	0.002
mod15	Age + Age:Dist K50 + AreaK95 + Breeding + Goat Sheep + Dist K50 + Dist Terr + (1∣Farm ID) + (1∣Bird ID) + (1∣Semester ID)	11	6797.61	11.070	0.002
mod16	Age + Age:Dist K50 + AreaK95 + Breeding + Dist K50 + Dist HPFP + Dist Road + Dist Terr + Sex + (1∣Farm ID) + (1∣Bird ID) + (1∣Semester ID)	13	6800.44	13.891	0.000
mod17	Age + Age:Dist K50 + AreaK95 + Dist K50 + Dist HPFP + Dist Road + Dist Terr + Sex + (1∣Farm ID) + (1∣Bird ID) + (1∣Semester ID)	12	6800.56	14.013	0.000
mod18	Age + Age:Dist K50 + AreaK95 + Breeding + Dist K50 + Dist HPFP + Dist Road + Dist Terr + Success + (1∣Farm ID) + (1∣Bird ID) + (1∣Semester ID)	13	6802.84	16.293	0.000
mod19	Age + Age:Dist K50 + AreaK95 + Breeding + Dist K50 + Dist HPFP + Dist Road + Dist Terr + (1∣Farm ID) + (1∣Bird ID) + (1∣Semester ID)	12	6802.98	16.434	0.000
mod20	Age + Age:Dist K50 + AreaK95 + Dist K50 + Dist HPFP + Dist Road + Dist Terr + Success + (1∣Farm ID) + (1∣Bird ID) + (1∣Semester ID)	12	6803.03	16.483	0.000
mod0	(1∣Farm ID) + (1∣Bird ID) + (1∣Semester ID)	4	8926.82	2140.272	0.000

Table S10. Top-ranked models assessing drivers of the selection of farms by Canarian Egyptian Vultures for the response variable *non-territorial VULTURES*. See Table 2 for a full description of explanatory variables. The number of parameters (K), the AICc, the difference in AICc between each model and the best model (ΔAICc), and Akaike weights (wi) are shown. "1|Bird ID", "1|Farm ID" and "1|Semester ID" indicate the random effect of bird, farm, and Semester identity respectively. Only the 20 highest-ranked and the null (intercept plus random terms) models are shown. All combinations from only the intercept to all considered fixed effects were tested.

Model	Variable	K	AICc	ΔAICc	wi
mod1	Age + AreaK95 + Breeding + Breeding:Dist Terr + Carcass + Goat Sheep + Dist K50 + Dist Road + Dist Terr + (1|Bird ID) + (1|Farm ID) + (1|Semester ID)	13	19838.19	0.000	0.316
mod2	AreaK95 + Breeding + Breeding:Dist Terr + Carcass + Goat Sheep + Dist K50 + Dist Road + Dist Terr + (1|Bird ID) + (1|Farm ID) + (1|Semester ID)	12	19838.28	0.091	0.302
mod3	Age + AreaK95 + Breeding + Breeding:Dist Terr + Goat Sheep + Dist K50 + Dist Road + Dist Terr + (1|Bird ID) + (1|Farm ID) + (1|Semester ID)	12	19839.15	0.959	0.196
mod4	AreaK95 + Breeding + Breeding:Dist Terr + Goat Sheep + Dist K50 + Dist Road + Dist Terr + (1|Bird ID) + (1|Farm ID) + (1|Semester ID)	11	19839.25	1.056	0.186
mod5	Age + AreaK95 + Breeding + Breeding:Dist Terr + Goat Sheep + Dist K50 + Dist Urb + Dist Terr + (1|Bird ID) + (1|Farm ID) + (1|Semester ID)	12	19893.72	55.529	0.000
mod6	AreaK95 + Breeding + Breeding:Dist Terr + Goat Sheep + Dist K50 + Dist Urb + Dist Terr + (1|Bird ID) + (1|Farm ID) + (1|Semester ID)	11	19893.87	55.681	0.000
mod7	Age + AreaK95 + Breeding + Breeding:Dist Terr + Carcass + Goat Sheep + Dist K50 + Dist Terr + (1|Bird ID) + (1|Farm ID) + (1|Semester ID)	12	19920.65	82.458	0.000
mod8	AreaK95 + Breeding + Breeding:Dist Terr + Carcass + Goat Sheep + Dist K50 + Dist Terr + (1|Bird ID) + (1|Farm ID) + (1|Semester ID)	11	19921.01	82.819	0.000
mod9	Age + AreaK95 + Breeding + Carcass + Goat Sheep + Dist K50 + Dist Road + Dist Terr + Sex + Sex:Dist Road + (1|Bird ID) + (1|Farm ID) + (1|Semester ID)	14	19924.97	86.782	0.000

Model	Variable	K	AICc	ΔAICc	wi
mod10	AreaK95 + Breeding + Carcass + Goat Sheep + Dist K50 + Dist Road + Dist Terr + Sex + Sex:Dist Road + (1 I Bird ID) + (1 I Farm ID) + (1 I Semester ID)	13	19925.26	87.075	0.000
mod11	Age + AreaK95 + Breeding + Goat Sheep + Dist K50 + Dist Road + Dist Terr + Sex + Sex:Dist Road + (1 I Bird ID) + (1 I Farm ID) + (1 I Semester ID)	13	19926.34	88.152	0.000
mod12	AreaK95 + Breeding + Goat Sheep + Dist K50 + Dist Road + Dist Terr + Sex + Sex:Dist Road + (1 I Bird ID) + (1 I Farm ID) + (1 I Semester ID)	12	19926.64	88.452	0.000
mod13	Age + AreaK95 + Carcass + Goat Sheep + Dist K50 + Dist Road + Dist Terr + Sex + Sex:Dist Road + (1 I Bird ID) + (1 I Farm ID) + (1 I Semester ID)	13	19928.43	90.238	0.000
mod14	AreaK95 + Carcass + Goat Sheep + Dist K50 + Dist Road + Dist Terr + Sex + Sex:Dist Road + (1 I Bird ID) + (1 I Farm ID) + (1 I Semester ID)	12	19929.47	91.279	0.000
mod15	Age + AreaK95 + Goat Sheep + Dist K50 + Dist Road + Dist Terr + Sex + Sex:Dist Road + (1 I Bird ID) + (1 I Farm ID) + (1 I Semester ID)	12	19929.79	91.599	0.000
mod16	AreaK95 + Goat Sheep + Dist K50 + Dist Road + Dist Terr + Sex + Sex:Dist Road + (1 I Bird ID) + (1 I Farm ID) + (1 I Semester ID)	11	19930.57	92.378	0.000
mod17	Age + AreaK95 + Breeding + Breeding:Dist Terr + Goat Sheep + Dist K50 + Dist Terr + (1 I Bird ID) + (1 I Farm ID) + (1 I Semester ID)	11	19931.35	93.156	0.000
mod18	AreaK95 + Breeding + Breeding:Dist Terr + Goat Sheep + Dist K50 + Dist Terr + (1 I Bird ID) + (1 I Farm ID) + (1 I Semester ID)	10	19931.74	93.554	0.000
mod19	Age + AreaK95 + Breeding + Carcass + Goat Sheep + Dist K50 + Dist Road + Dist Terr + Sex + Sex:areaK95 + (1 I Bird ID) + (1 I Farm ID) + (1 I Semester ID)	14	19932.70	94.507	0.000
mod20	AreaK95 + Breeding + Carcass + Goat Sheep + Dist K50 + Dist Road + Dist Terr + Sex + Sex:areaK95 + (1 I Bird ID) + (1 I Farm ID) + (1 I Semester ID)	13	19933.30	95.111	0.000
mod0	(1 I Bird ID) + (1 I Farm ID) + (1 I Semester ID)	4	23521.86	3683.668	0.000
