# Supplementary material for: Vultures and Livestock: The Where, When, and Why of Visits to Farms
Source: Animals (Basel). 2020 Nov 16;10(11):2127. doi: 10.3390/ani10112127 (PMC7698296; doi:10.3390/ani10112127)

Spline correlograms (top) and values of Moran's I (bottom) for top-ranked models determining the use of farms by Canarian Egyptian Vultures on Fuerteventura. Distance is in metres, k indicates number of neighbouring farms. For *VULTURES* variables, model residuals used for calculating spline correlograms correspond to mean values weighted by Akaikes weights.

Figure S4. Results for model of the response variable *FARMS*.

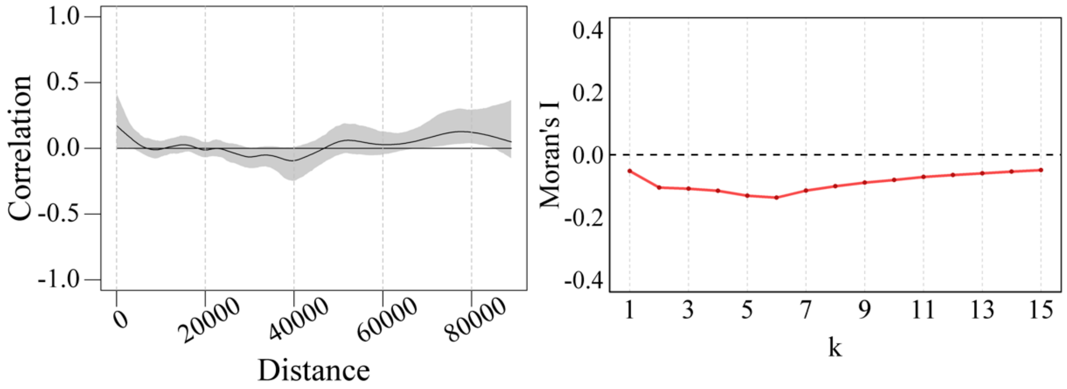

Figure S5. Results for models of the response variable *non-territorial VULTURES*.

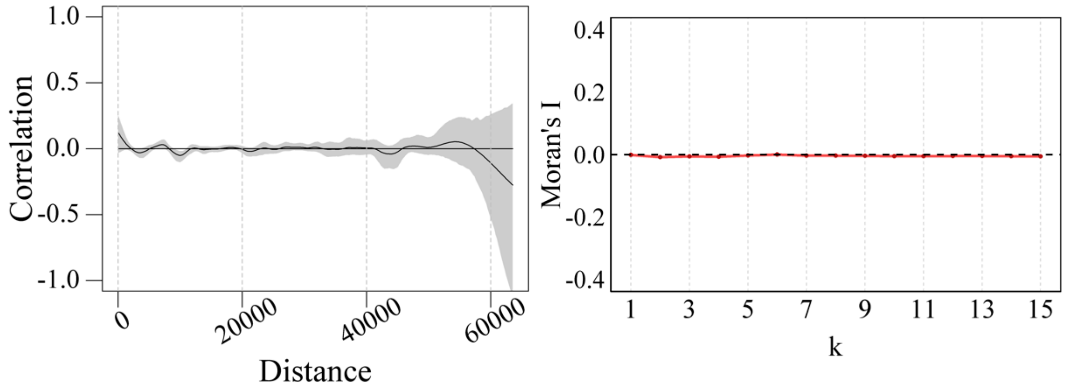

Figure S6. Results for models of the response variable *territorial VULTURES*.

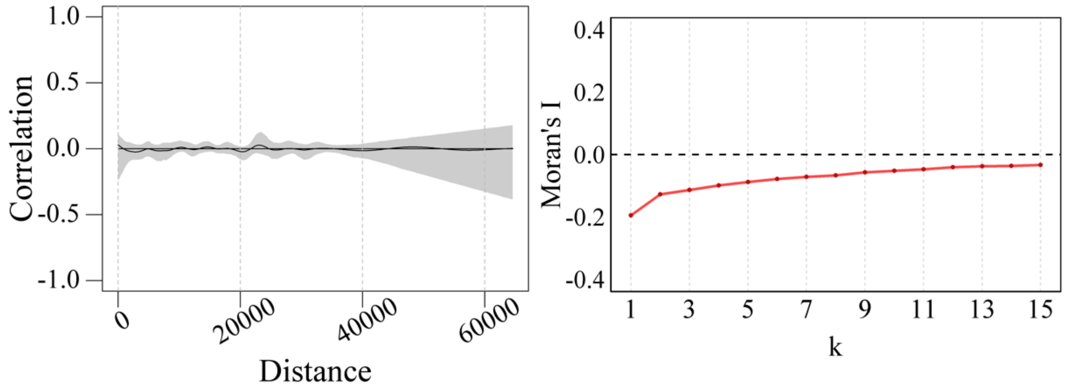

Supplement: Supplementary file 1 [file animals-10-02127-s001.zip › supplementary 9_Figure S4-6.pdf]
